# Developmental changes in the morphology of mouse hypoglossal motor neurons

**DOI:** 10.1007/s00429-015-1130-8

**Published:** 2015-10-17

**Authors:** Refik Kanjhan, Matthew J. Fogarty, Peter G. Noakes, Mark C. Bellingham

**Affiliations:** 1 School of Biomedical Sciences, University of Queensland, Brisbane, QLD 4072 Australia; 2 Queensland Brain Institute, University of Queensland, Brisbane, QLD 4072 Australia

**Keywords:** Axon collateral, Dendritic morphology, Dye-coupling, Postnatal development, Respiration, Spine

## Abstract

Hypoglossal motor neurons (XII MNs) innervate tongue muscles important in breathing, suckling and vocalization. Morphological properties of 103 XII MNs were studied using Neurobiotin™ filling in transverse brainstem slices from C57/Bl6 mice (*n* = 34) from embryonic day (E) 17 to postnatal day (P) 28. XII MNs from areas thought to innervate different tongue muscles showed similar morphology in most, but not all, features. Morphological properties of XII MNs were established prior to birth, not differing between E17–18 and P0. MN somatic volume gradually increased for the first 2 weeks post-birth. The complexity of dendritic branching and dendrite length of XII MNs increased throughout development (E17–P28). MNs in the ventromedial XII motor nucleus, likely to innervate the genioglossus, frequently (42 %) had dendrites crossing to the contralateral side at all ages, but their number declined with postnatal development. Unexpectedly, putative dendritic spines were found in all XII MNs at all ages, and were primarily localized to XII MN somata and primary dendrites at E18–P4, increased in distal dendrites by P5–P8, and were later predominantly found in distal dendrites. Dye-coupling between XII MNs was common from E18 to P7, but declined strongly with maturation after P7. Axon collaterals were found in 20 % (6 of 28) of XII MNs with filled axons; collaterals terminated widely outside and, in one case, within the XII motor nucleus. These results reveal new morphological features of mouse XII MNs, and suggest that dendritic projection patterns, spine density and distribution, and dye-coupling patterns show specific developmental changes in mice.

## Introduction

Hypoglossal (XII) motor neurons (MNs) are located in bilaterally paired XII motor nuclei in the dorsomedial medulla oblongata, and control the activity of several extrinsic and intrinsic tongue muscles that take part in a number of basic and important motor functions, including mastication, swallowing, suckling, vocalization and respiration (Fregosi 2011; Aldes 1995; Altschuler et al. 1994; Sokoloff 1993; Berger et al. 1995). Understanding how XII MN morphology contributes to the generation and control of XII motor activity is critical, both for comprehending these functions and for a better understanding of how dysfunction or death of XII MNs contributes to several diseases. These include amyotrophic lateral sclerosis (DePaul et al. 1988; van Zundert et al. 2008), obstructive sleep apnea (Dempsey et al. 2010), Rett syndrome (Voituron et al. 2010) and sudden infant death syndrome (Kinney 2009). While the electrophysiology of synaptic and neurochemical responses of XII MNs has been intensively studied (Rekling et al. 2000), much less attention has been paid to the dendritic structure of individual XII MNs. Previous studies have shown that dendritic structures impose a fundamental constraint on the location and integration of synaptic inputs (Rall 1959; Häusser et al. 2000).

Retrograde neuronal tracers applied by topical application to the proximal nerve stump or by intramuscular injections or chromatolysis of motor neurons following nerve section have revealed a consistent myotopic or somatotopic organization of XII MNs within the XII motor nucleus of several species, including the mouse: a dorsal subdivision innervating the tongue retrusor hyoglossus and styloglossus muscles; a ventromedial subdivision innervating the tongue protrusor genioglossus muscle; and a ventrolateral subdivision innervating the suprahyoid protrusor geniohyoid muscle (Krammer et al. 1979; Altschuler et al. 1994; Aldes 1995; Uemura Sumi et al. 1988; Sokoloff 1993; Stuurman 1916). This segregation of XII MNs innervating different tongue muscles is already established at birth, and persists throughout postnatal development into adulthood (Sokoloff 1993). Of the several tongue muscles innervated by the XII nerve, the extrinsic genioglossus muscle has been most intensively studied, and exhibits rhythmic inspiratory activity in conjunction with basal tonic activity (Fregosi and Fuller 1997; Fregosi 2011). The genioglossus is therefore thought to play an important role in maintenance of upper airway patency, as the protrusive anterior movement of the tongue caused by genioglossus activation widens the upper airway (Fregosi 2011). Most notably, reduced inspiratory and tonic genioglossus activity is thought to be an important contributing factor to the pathogenesis of obstructive sleep apnea (Wheatley et al. 1993; Horner 2007; Saboisky et al. 2007; Remmers et al. 1978). However, other intrinsic and extrinsic tongue muscles innervated by the XII nerve also show respiratory activity and can contribute to the maintenance of upper airway patency (Bailey and Fregosi 2004; Fregosi 2011; Mateika et al. 1999). Despite such functional importance, we do not know whether XII MNs innervating the various tongue muscles are a relatively uniform population of efferent neurons or, if not, how they differ from each other.

Dendrites of retrogradely or individually dye-filled XII MNs have been shown to branch extensively, often expanding into the surrounding reticular formation around the lateral and ventral sides of the XII nucleus, and with some dendrites crossing the midline to the contralateral XII nucleus [for cat (Withington-Wray et al. 1988; Altschuler et al. 1994), rat (Núñez-Abades et al. 1994; Koizumi et al. 2008; Wan et al. 1982) and mouse (Tarras-Wahlberg and Rekling 2009; van Zundert et al. 2008)]. These commissural dendritic crossings have been shown to be developmentally regulated in the rat and mouse, normally disappearing between postnatal days 8 and 18 (van Zundert et al. 2008; Núñez-Abades et al. 1994), reappearing again at P19, and then persisting into adulthood in the rat (Núñez-Abades et al. 1994). The extensive dendritic arborization of XII MNs provides potential postsynaptic sites for integration of premotor synaptic inputs and synchronization of respiratory neuronal activity (Altschuler et al. 1994; Núñez-Abades et al. 1994; Tarras-Wahlberg and Rekling 2009; Koizumi et al. 2008). It is therefore important to understand whether XII MNs innervating different tongue muscles show similar morphological features and, in particular, the patterns of XII MN dendrite branching and degree to which dendrites extend beyond the borders of the XII motor nucleus containing the MN soma, as these morphological features may preclude or allow integration of synaptic inputs from different sets of ipsilateral and contralateral premotor neurons.

In vitro and in vivo studies have shown that rhythmic inspiratory XII nerve activity is primarily due to excitatory synaptic drive by glutamatergic synapses (Funk et al. 1993, 1997a; Steenland et al. 2006, 2008). Immunohistochemical studies show that rodent XII MNs receive numerous glutamatergic synaptic terminals (O’Brien et al. 1997; Travers et al. 2005; Fogarty et al. 2013). Trans-synaptic retrograde viral tracing studies in rodents consistently show that premotor interneurons for XII MNs lie in the reticular formation lateral and ventrolateral to the XII motor nucleus [for rat (Dobbins and Feldman 1995; Card et al. 1990; Fay and Norgren 1997; Chamberlin et al. 2007; Ugolini 1995); for mouse (Babic et al. 1993; Ugolini 1995; Ugolini et al. 1987)]. Many of these interneurons are likely to be glutamatergic, as short latency glutamatergic EPSPs are evoked in XII MNs following electrical stimulation in the reticular formation lateral and ventral to the border of the XII nucleus in rat (Bellingham and Berger 1994, 1996; Bellingham 2013) and mouse (Ireland et al. 2004, 2012). Furthermore, neurons in this premotor region can be antidromically activated or retrogradely labeled from the XII nucleus in mouse, rat and cat (Koizumi et al. 2008; Bellingham and Berger 1996; Woch et al. 2000; Chamberlin et al. 2007; Peever et al. 2002; Ono et al. 1994; Li et al. 1993; Tarras-Wahlberg and Rekling 2009; Holstege et al. 1977).

Inspiratory premotor interneurons in the lateral and ventral reticular formation provide excitatory synaptic inputs to rat XII MNs (Peever et al. 2002; Koizumi et al. 2008). Exogenous activation of glutamatergic receptors markedly increases XII MN membrane conductance (Bellingham and Berger 1996; Rekling et al. 2000), but endogenous synaptic activation of glutamate receptors during rhythmic inspiratory depolarization produces relatively small changes in membrane conductance measured at the soma (Funk et al. 1997b; Ramirez et al. 1996). It is therefore likely that the site of glutamatergic inputs from these inspiratory premotor interneurons to XII MNs is predominantly on distal dendrites. However, the distribution of glutamatergic inputs onto the dendrites of XII MNs, and how this might change during development, remains poorly characterized.

The morphology of individual genioglossus XII MNs filled with Neurobiotin or horseradish peroxidase has been described in rat (Mazza et al. 1992; Núñez-Abades and Cameron 1995; Núñez-Abades et al. 1994; Koizumi et al. 2008), guinea pig (Viana et al. 1990; Mosfeldt Laursen and Rekling 1989) and cat (Withington-Wray et al. 1988). Currently, there are no systematic studies of single XII MN morphology in the mouse during development, and it is not known whether the morphological properties and developmental changes of XII MNs in the mouse are similar to those seen in the rat or cat (Mazza et al. 1992; Núñez-Abades and Cameron 1995; Núñez-Abades et al. 1994). There is a strong need for this knowledge, as studies in recent years have been increasingly using mice as the species of choice, due to the ready availability of genetically modified mouse strains (van Zundert et al. 2008; Banks et al. 2005; Voituron et al. 2010). Here, we have systematically studied the developmental alteration of the morphological properties of single Neurobiotin-filled XII MNs from all regions of the XII motor nucleus, and from late embryonic ages up to 28 days post-birth, when functional maturity has been reached. This work was done in the C57Bl6 inbred mouse strain, as this strain is commonly used in generation of transgenic and gene targeted mutant mice.

## Experimental procedure

### Ethical approvals

All experiments were performed in accordance with the Australian Code of Practice of the National Health and Medical Research Council (7th edition, 2004) and the EC Directive 86/609/EEC for animal experiments; procedures and animal use were approved by the University of Queensland Anatomical Biosciences Animal Ethics Committee (approval number SBMS/093/09). Reporting of experimental results complied with the uniform requirements for manuscripts submitted to *Biomedical Journal* (http://www.icmje.org), and with policies and regulations regarding animal experimentation and other ethical matters (Drummond 2009).

### Experimental animals used

The XII motor neurons described here were obtained from 34 C57Bl6 mice from 24 different litters. The sex of mice less than 5 days old (*n* = 14 animals) was not determined, as anogenital distance is not a reliable guide to sex in this age range; XII motor neurons in mice 5 days or older (*n* = 20 animals) came from 10 males and 10 females, with sex being determined by the presence of inguinal teats in females (Greenham and Greenham 1977). The number and ages of mice and the number of litters used in each age group were: E17–P0 [6 mice (1 at E17, 2 at E18, 3 at P0), 6 litters], P1–4 [8 mice (2 at P1, 1 at P2, 2 at P3, 3 at P4), 8 litters], P5–8 [6 mice (1 at P5, 1 at P6, 2 at P7, 2 at P8), 6 litters], P9–13 [5 mice (1 at P9, 2 at P10, 2 at P13), 4 litters] and P14–28 [9 mice (1 at P14, 1 at P15, 1 at P16, 1 at P17, 1 at P19, 1 at P21, 1 at P24, 1 at P27, 1 at P28), 6 litters]; animals from the same litter were killed at different ages.

### Surgical and experimental procedures

Embryonic pups were surgically obtained, following euthanasia of the mother by cervical dislocation. Embryonic (E17–18) and newborn pups (P0–3) were anesthetized by hypothermia for ~3 min, while older mice were deeply anesthetized by intraperitoneal injection of sodium pentobarbitone (60–80 mg/kg, Vetcare, Brisbane, Australia). Following removal of skin and underlying bones, and decerebration rostral to the pons, the animals were dipped in ice-cold modified (high-Mg^2+^) Ringer solution. The modified high-Mg^2+^ ringer solution for E17–P14 ages contained (in mM): 130 NaCl, 3 KCl, 26 NaHCO_3_, 1.25 NaH_2_PO_4_, 5 MgCl_2_, 1 CaCl_2_, and 10 d-glucose. For animals older than P14, we used a high-sucrose-Mg^2+^ Ringer solution with iso-osmotic replacement of NaCl by sucrose, containing (in mM): 218 sucrose, 3 KCl, 26 NaHCO_3_, 1.25 NaH_2_PO_4_, 5 MgCl_2_, 1 CaCl_2_, and 10 d-glucose (osmolarity ~300 mOsm, Vapro 5520 osmometer, Wescor, South Logan, UT, USA) (Bellingham and Berger 1996). All Ringer solutions were continuously bubbled with 95 % O_2_/5 % CO_2_ to maintain pH at 7.4. The preparation was pinned down from dorsal side up in a large dissection bath filled with ice-cold ringer slurry. The brainstem and the cervical spinal cord were then rapidly dissected from the surrounding tissues by a laminectomy and sectioning nerve rootlets with the aid of a Carl Zeiss dissection microscope. The tissue block was fixed rostral end down with superglue onto a holding metal bath, the ventral side rested on an agar block previously superglued to the metal bath, which was then filled with the same ice-cold Ringer solution used in dissection. Transverse brainstem sections (300 μm thick) were cut using a vibratome (DSK 1000, Ted Pella Inc. CA, USA), which had a large water bath kept ice-cold surrounding the metal bath. During sectioning, bilateral hypoglossal nuclei could usually be identified with a dissection microscope, as darker spots located ventral and lateral to the central canal or the 4th ventricle on either side of the midline. Usually four serial sections from caudal end to the rostral end containing XII nucleus were obtained per animal. The most caudal section had the central canal close to the center of the section, with the bundles of internal arcuate fibers separating the ventrolateral subnucleus from the main XII nucleus (Aldes 1995). The following two slices were marked as median sections as they had larger bilateral XII nuclei with more dorsally located central canal than the caudal section. At the rostral end of the median sections the central canal usually became the 4th ventricle. The most rostral section had no central canal and the bilateral XII motor nucleus occupied a smaller volume.

Cut slices were then moved from ice-cold bath solution (high-Mg^2+^ or high-sucrose-Mg^2+^) and incubated for 45 (<P14) to 60 (>P14) minutes in the same solution kept at 34 °C in a water bath. The sections were then moved to a normal Ringer solution in a similar incubation chamber and kept at room temperature (21–22 °C) for at least 30 min prior to start of recording and labeling. Normal Ringer solution used for recording in all ages contained (in mM): 130 NaCl, 3 KCl, 26 NaHCO_3_, 1.25 NaH_2_PO_4_, 1 MgCl_2_, 2 CaCl_2_, 10 d-glucose, and continuously bubbled with 95 % O_2_/5 % CO_2_ to maintain pH at 7.4. Slices were typically viable for Neurobiotin electroporation for ~6–8 h after this final transfer.

The electrodes were pulled from borosilicate glass capillaries (Vitrex Modulohm, Edwards Medical, NSW Australia) and were filled with pipette solution (giving a tip resistance of ~3–4 MΩ). The pipette solution contained 2 % Neurobiotin™ (NB, Vector Labs, Burlingame CA, USA) in an artificial intracellular solution containing 135 mM K^+^ (or Cs^+^)—methanesulphonate (MeSO_4_), 6 mM KCl, 1 mM EGTA [ethylene glycol bis (2-aminoethyl ether)-*N*,*N*,*N*′,*N*′-tetraacetic acid], 2 mM MgCl_2_, and 5 mM Na-HEPES (Na-4-2-hydroxyethyl-1-piperazineethanesulfonic acid), 3 mM ATP-Mg^2+^, 0.3 mM GTP-Tris (pH 7.25 with KOH, osmolarity of 305 ± 5 mOsm) (Kanjhan and Vaney 2008). This Neurobiotin-containing solution was placed in the pipette tip with a Hamilton syringe (1–2 μl) and the pipette was then backfilled with the same solution without Neurobiotin, to a level that contacted the coated silver electrode wire.

### Visualizing and recording XII MNs in brain slices

A single brainstem slice was placed in a tissue chamber and stabilized with the aid of a metal mesh on a Zeiss Axioskop 2 FS microscope fitted with IR-DIC optics, and continuously superfused with the normal Ringer solution at room temperature (22–24 °C). The tissue and recording electrodes were viewed on a Sony monitor through a 40× water-immersion objective and a 2× relay attachment located in front of the infrared video camera (WAT-902H, Watec Co. Ltd., Japan) mounted above the camera port of the microscope. XII MNs were typically found in distinct bilaterally located nuclei immediately ventral to the central canal at caudal levels or the 4th ventricle at rostral levels.

### Neurobiotin electroporation

Recordings and electroporation (controlling the amplitude and the duration of the voltage pulses) were made with an Axopatch 1D amplifier (Axon Instruments, Foster City, CA, USA). Data were acquired at a sampling rate of 5 or 10 kHz, low-pass filtered at 2 kHz and stored on a Macintosh G3 computer using Axograph 4.9 software and a Digidata 1332A digitizer (Axon Instruments).

XII MNs were randomly selected from the visually identified cells in several areas of the motor nucleus. We filled up to four cells in each slice: two on each side at dorsal and ventromedial portions of the nucleus whenever the hypoglossal motor nucleus had a large area. For most caudal and most rostral sections, where the area of the XII motor nucleus was small, only single MNs on each side of the medulla were filled. The number of individual motor neurons filled from an individual mouse ranged from 1 to 8; the number of MNs filled per mouse (with mouse age) was 11 mice (E17, P0, P0, P1, P3, P5, P5, P15, P16, P17, P28) with 1 MN, 8 mice (P3, P8, P8, P10, P10, P14, P19, P27) with 2 MNs, 6 mice (P1, P3, P3, P4, P9, P24) with 3 MNs, 3 mice (E18, P13, P15) with 4 MNs, 4 mice (E18, P4, P13, P21) with 5 MNs, 3 mice (P0, P2, P7) with 6 MNs, and 1 mouse (P6) with 8 MNs. This arrangement allowed tracing of individual MNs and identifying dye-coupled cells, as well as an unbiased selection of MNs that innervate different muscle groups.

Individual XII MNs were targeted visually and a patch-electrode was advanced towards the cell body with the aid of a manipulator (MPC-200, Sutter Instrument Company). A brief positive internal pressure sufficient to keep the tip clear was only applied during penetration into the tissue, to minimize tip blockage. The electrode tip was gently and rapidly pushed against the XII MN soma, and then a continuous gentle suction was applied until a stable seal of preferably >30 MΩ was obtained.

XII MNs usually did not show any spontaneous spiking when recordings were done with electrodes filled with the bath (normal Ringer) solution in semi-loose seal (50–300 MΩ) or tight-seal (>1 GΩ) configurations. However, when recordings were made with electrodes filled with K^+^-based artificial intracellular solutions, XII MNs sometimes displayed varying levels of spontaneous spiking that could be seen with semi-loose seal configuration (Kanjhan and Bellingham 2013).

**Fig. 1:**
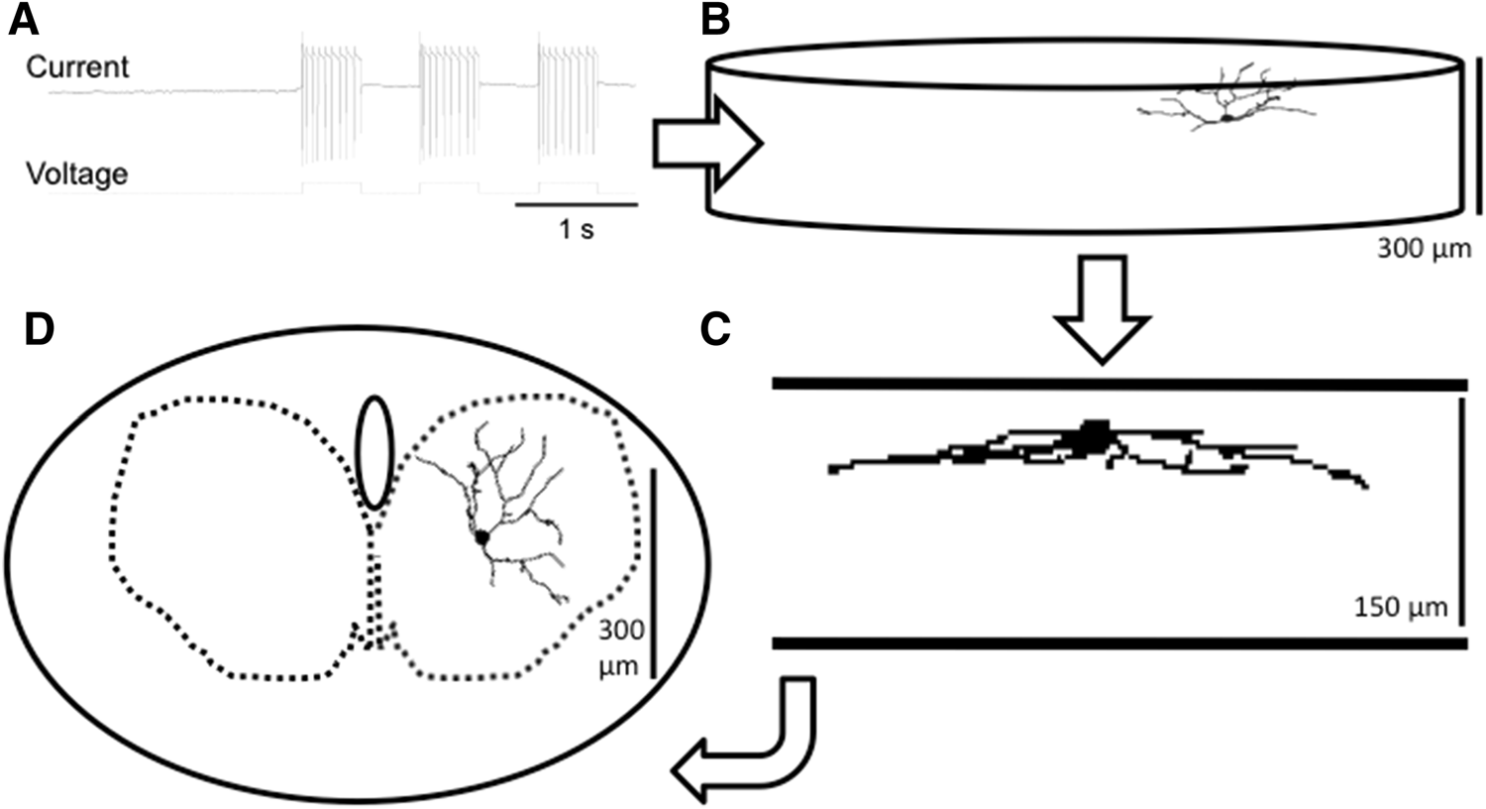
Schematic illustration of an electrophysiological recording protocol for semi-loose seal Neurobiotin electroporation (**a**), with the labeled XII MN firing action currents (downward deflections of the current trace) during electroporation by square voltage steps (50 mV steps of 500 ms duration, voltage trace) which depolarize the MN and cause repetitive firing. After recording, electroporation and fixation, the subsequent immunofluorescence reaction produces recovery of a single labeled HM, whose soma is located within 50 μm of the surface of the transverse brainstem tissue slice used for recording. This is illustrated as a flat cylinder at low magnification in **b** and shows a transverse orientation of its full dendritic arborization that is complete within the slice. The cell is shown at higher magnification in the sagittal plane in **c** and in the traverse plane in **d**. The step-like changes in dendrite morphology in **c** are due to the finite step size of the confocal stack used for morphological reconstruction with Neurolucida software. The completely reconstructed dendritic arbor of the same XII MN in **b** and **c**, shown projected onto the transverse plane in **d**, is entirely contained within the boundaries of the ipsilateral hypoglossal motor nucleus (*dashed outline*)

The semi-loose seal Neurobiotin electroporation procedure was modified from that described for retinal ganglion cells (Kanjhan and Vaney 2008). Square-wave voltage steps (0.5 s, 1 Hz) of 5–25 mV that generated current pulses of 300–500 pA were typically applied for 5–6 min (Fig. 1a, lower trace) (Kanjhan and Bellingham 2013). In the semi-loose configuration, the cell membrane usually breaks down easily in the presence of Neurobiotin in the pipette solution (Kanjhan and Vaney 2008). Because the semi-loose seal method is less disruptive of the neuronal membranes, neuron survival rates after pulling the recording electrode away from the neuron were very high, and these properties together allowed serial recording and labeling of many cells from the same slice preparation. Using this method, our success rate of recovering Neurobiotin-electroporated XII MNs was ~95 %.

If a tight-seal formed during suction through the pipette, then the cell was stimulated with 50–100 mV square-wave pulses of 500 ms duration at 1 Hz, until these caused a significant drop in the membrane input resistance (e.g., ~80 MΩ), which usually occurred within 10–30 s. This signaled a breakdown of the membrane at the pipette tip. As soon as the membrane broke, as indicated by a drop in resistance, the voltage steps were stopped, and synaptic currents could be recorded at desired holding potentials. In the tight-seal configuration, the baseline shift was close to resting membrane potential (–50 to –65 mV) and the spike amplitude was ~80–90 mV. The pipette solution had to contain K^+^-based artificial intracellular solution to record action potentials in current-clamp mode (Kanjhan and Bellingham 2013).

In this mode, the cells were also filled simply by diffusion of Neurobiotin through the pores formed during electroporation, given that the recording configuration lasted for at least 20 min. To be sure of adequate labeling, we often stimulated a further 3–5 min with current pulses of 300–500 pA amplitude to fill the cells with Neurobiotin at the completion of recording. Whole-cell patch clamping with dye-containing electrodes have previously been used to fill cells in brain slices, usually limited to one or two cells. In our experience, the difficulties with tight-seal formation and the damage caused during electrode pull-out reduced the success rate and limited the number of cells filled with the whole-cell technique. About 35 % of cells with tight-seal configuration were either not recovered at all, or, if recovered, they displayed substantial damage to cell soma and/or processes, such as vacuolated or segmented appearance of dendrites and/or missing cell soma. These cells were not analyzed in the data set presented here.

### Post-recording tissue processing and visualization

Upon completion of recording and labeling, the sections were left resting in the bath for a minimum of 10 min to allow diffusion of Neurobiotin throughout the XII MN. The sections were then fixed in 4 % paraformaldehyde in 0.1 M phosphate buffer (pH 7.4) for 20–30 min, and subsequently washed 3–4 times for several hours in 0.1 M phosphate-buffered saline (PBS). Subsequently sections were placed for a minimum of 2 h in PBST (PBS with added 0.05 % Triton-X100) containing 2 % bovine serum albumin (BSA) to reduce non-specific background. Later, the sections were incubated for 2–4 h in Cy3-Streptavidin (Sigma Aldrich, Castle Hill, Australia, 1:500 in PBST with 2 % BSA). The sections were washed overnight and then individually mounted on uncoated glass slides using a glycerol-based *p*-phenylenediamine mounting medium (10 mg *p*-phenylenediamine in 9 ml of glycerol plus 1 ml of 0.1 M phosphate buffer). Small glass cover slips of ~10 mm × 10 mm cut with a diamond knife were used to reduce the amount of pressure they put onto brain slices. The edges of the coverslips were sealed with nail polish. The sections were imaged with a Zeiss LSM 510 Meta confocal microscope.

### Morphometric measurements and statistical analysis

Morphological properties of 103 XII MNs filled with Neurobiotin were analyzed from stacks of confocal images obtained at low and high magnifications (from 7× to 157.5×). All the XII MN cell bodies were located within the borders of the XII motor nuclei located bilaterally in the dorsomedial medulla, immediately ventral and lateral to the central canal at the caudal end or the 4th ventricle at the rostral end of the hypoglossal motor nucleus (e.g., Fig. 1d). Neurobiotin was confined to the soma and dendrites of the recorded and filled cell, with negligible spillover in the extracellular space (Fig. 1c, d). Subsequently, morphological properties (dendritic projection, branching and length, dendritic spines, dye-coupling) of filled cells were analyzed using Neurolucida™ software (MBF Bioscience Inc, Williston, VA, USA). Briefly, dendritic lengths were measured by tracing the entire arborization in the three-dimensional confocal stack at low objective magnifications (20×) (Fogarty et al. 2013). Detailed analysis of dendritic spines was performed by tracing them through serial confocal stacks at high objective magnification (63×) (Fogarty et al. 2013). Dendritic processes were classified as spines only if they were no greater than 6 µm in length and no greater than 0.8 µm in cross-sectional diameter (Fogarty et al. 2013), to be consistent with other morphometric spinal parameters describing dendritic spines as being less than 0.8 µm^3^ (Harris and Kater 1994; Harris 1999). Soma surface area and volume were directly measured from the confocal stack images by Neurolucida™ software (Bitplane, South Windsor, CT, USA). Dendrite surface area and volume were calculated from the length and radius of dendritic cylinders (Microsoft Excel, Microsoft, Redmond, WA, USA).

Prism 6.0 (Graphpad, Sorrento, CA, USA) was used to calculate the mean values of all measurements and for all statistical analysis. Data are presented as means ± 95 % confidence intervals (CI) except where indicated; and significance was accepted at the *P* < 0.05 level. Multiple age groups were compared using one or two-way ANOVA tests where specified, with inter-group comparisons made using Tukey’s multiple comparison (one-way ANOVA) or Bonferroni’s multiple comparison (two-way ANOVA). Linear regression was done in Prism, as were all other statistical tests used, where indicated in the results.

All chemicals were obtained from Sigma Aldrich (Castle Hill, NSW, Australia) except where otherwise indicated.

## Results

### Location of filled XII MNs

**Fig. 2:**
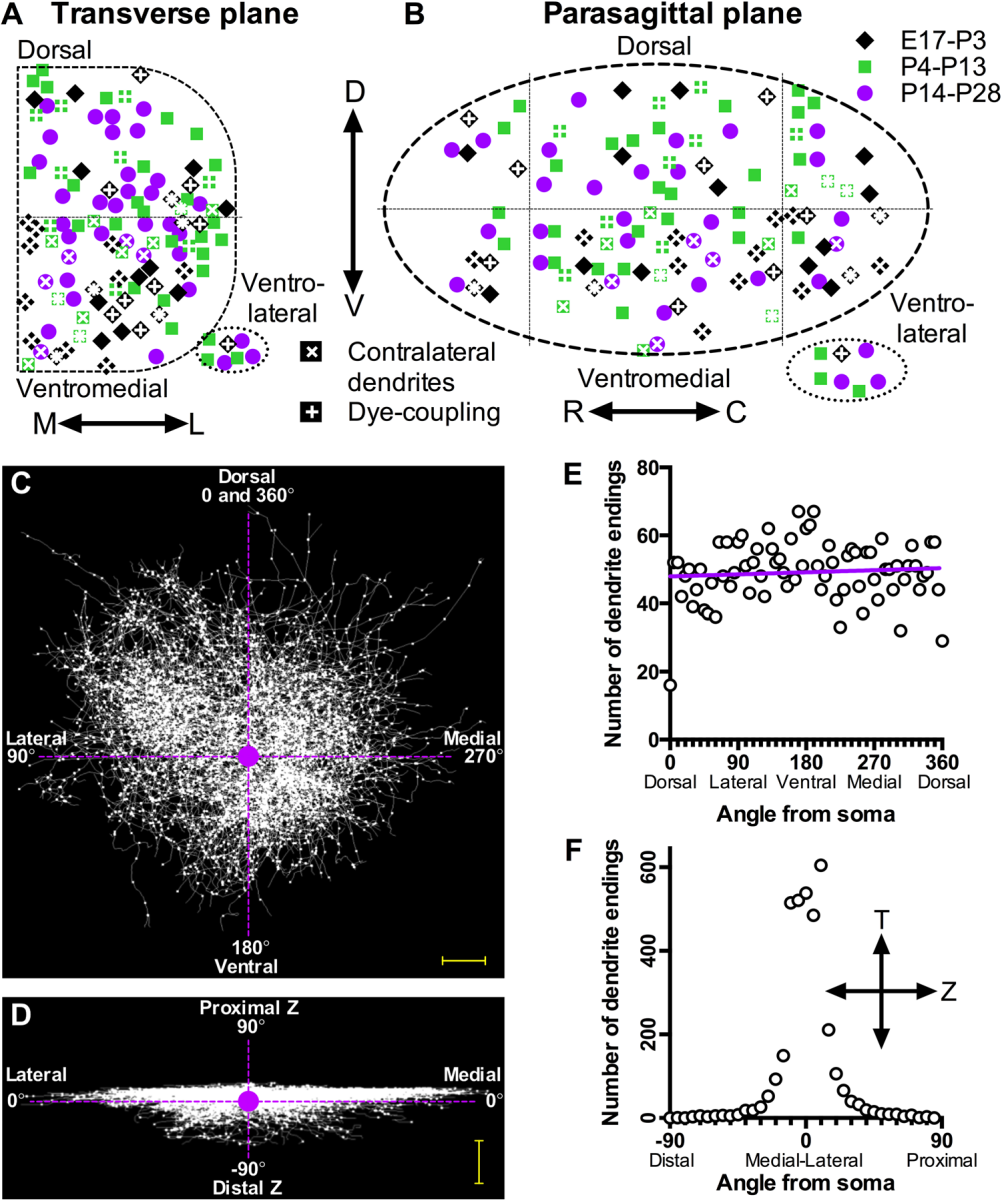
Location within the hypoglossal motor nucleus and characteristics of individually labeled XII motor neurons from mice aged E17–P28. **a** Schematic outline of the right hypoglossal motor nucleus in the transverse plane, divided into dorsal, ventromedial and ventrolateral sectors; the projected location of each recorded XII MN is shown by symbols according to age group (E17–P3 as *diamond*, P4–13 as *square*, P14–28 as *circle*), while symbols with a superimposed white ‘+’ denote XII MNs with dye-coupling and symbols with a superimposed white ‘×’ denote XII MNs with dendrites crossing the midline to the contralateral brainstem. **b** Schematic outline of the right hypoglossal motor nucleus in the parasagittal plane, divided into dorsal and ventral sectors and rostral/median/caudal sectors by dashed lines, with all labeled XII MNs from (**a**) projected onto their rostro-caudal position within the motor nucleus. **c** Transverse view of the superimposed wire filament reconstructions of the dendrites of all XII MNs in the study, radiating from a single point (the MN soma, *purple*/*filled circle* in center **c** and **d**). **d** The same superimposed wire filament reconstructions of all XII MNs in the study, in a view which is orthogonal to that of **c**; it can be noted that the rostro-caudal projections of dendrites (noted by the proximal and distal z positions) is very restricted in XII MNs. **e** Plot of the number of dendritic terminal points (end of each dendritic branch) which terminated within successive polar sectors of 5° around the soma of all XII MNs in the study in the transverse plane; the data have been fitted with a *straight line* by linear regression (*dashed line*), whose slope was not significantly different to 0. **f** Similar plot of the dendritic terminal points in the sagittal plane of all XII MNs in the study; by contrast to the uniformly random terminal positions in the transverse plane (**e**), the distribution of dendrite terminals in the rostro-caudal axis is spatially restricted in XII MNs. *Scale bars* **c** and **d** = 100 µm

Figure 2a, b summarizes the relative locations of all filled XII MNs (*n* = 103) from all ages (E17–P28; see “Results”, see “Experimental animals used” for animal ages sampled) analyzed in this study. Four 300-μm-thick transverse slices containing the XII motor nucleus were obtained from each animal and each slice was marked as caudal, median-caudal, median-rostral, and rostral. The dorso-ventral and medio-lateral coordinates of each filled XII MN were measured relative to the boundaries of the XII motor nucleus in individual brainstem slices. The rostro-caudal position of each slice was determined by comparison of slice landmarks to a mouse brain atlas (Franklin and Paxinos 2008) to provide a rostro-caudal coordinate for XII MNs recorded in each slice. The dorso-ventral and medio-lateral coordinates for each XII MN were projected onto a standard D-shaped area representing the transverse boundary of a single XII motor nucleus (Fig. 2a), while the rostro-caudal and dorso-ventral coordinates for each XII MN were projected onto an elliptical area representing the parasagittal boundary of a single XII motor nucleus (Fig. 2b). Figure 2a, b thus shows that XII MNs were sampled from all areas thought to supply the different intrinsic and extrinsic tongue muscles innervated by the XII nerve.

### General features of XII MN dendrite projections

The dendritic projections of individual XII MNs from different parts of the XII motor nucleus projected to four to five overlapping fields in the dorsal, dorsomedial, lateral, ventral and ventromedial directions (Fig. 2c, e). The majority of the dendritic structure of individual XII MNs was oriented in the transverse plane (Altschuler et al. 1994), and thus was retained within the brainstem slice (see example shown in Fig. 1b, c). This is readily seen in Fig. 2c, d, in which a computer-generated wire trace of the dendritic structure of each XII MN used in this study has been projected onto a composite whole with the MN somas as a common center (shown as a central purple circle). Figure 2c shows the composite dendritic projection pattern in the transverse plane, while Fig. 2d shows the same composite in the orthogonal plane (90° to the transverse plane), illustrating the predominant transverse projections of dendrites with relatively little dendritic projection in the rostro-caudal direction (indicated by Proximal Z and Distal Z in Fig. 2d). Cells with cut off dendrites could be identified, as the cell body was always located 10–40 μm below the surface of the slice, and cells with significant dendritic loss (i.e., missing dendritic projections in any cardinal transverse directions of dorsal, medial, lateral and ventral) were not included in the analysis.

If dendrites tended to project in a particular direction, then there should be a non-random radial distribution of the terminal ends of dendrites. To test this, the position of the terminal ends of each dendrite were binned into radial sectors of 5° around the soma to quantify the number of dendrites terminating in all sectors. For the transverse plane, Fig. 2e shows the number of dendrites in each radial sector, plotted on a linear scale; linear regression (indicated by the purple line in Fig. 2e) found that slope was not significantly different from zero (*P* = 0.49, *F* = 0.4720, *df* = 71) and a runs test was also not significantly different from linearity (*P* = 0.35). By contrast, in a similar plot for the orthogonal plane (Fig. 2f), while linear regression slope was not significantly different from zero (*P* = 0.81, *F* = 0.054, *df* = 34; fitted line not shown for clarity), a runs test was highly significantly different from linearity (*P* < 0.0001).

XII MNs from different parts of the XII motor nucleus did not differ consistently in their morphological features (as detailed below), with two notable exceptions. Firstly, XII MNs with dendrites crossing the midline (symbols with superimposed white ‘×’ in Fig. 2a, b, see below for further analysis and the example shown in Fig. 9b) were almost exclusively found in the ventromedial XII motor nucleus. Secondly, dendrites of XII MNs located in the ventrolateral XII motor nucleus did not cross to the contralateral side. Dye-coupled XII MNs (indicated with a superimposed white ‘+’ symbol in Fig. 2a, b, see below) were widely distributed throughout the XII motor nucleus.

The dendrites of XII MNs largely elaborated within the XII motor nucleus (see examples shown in Fig. 5a–d). Distal dendrites of a minority (23.3 %, 24 of 103 XII MNs) sometimes extended beyond the lateral XII motor nucleus boundaries (excluding the medial midline boundary). For this minority of XII MNs, the mean % of total dendrite length lying outside the XII motor nucleus boundary was 12.3 % (95 % CI of 8.3–16.3 %). These distal dendritic processes extended into the adjacent reticular formation in the dorsomedial, dorsolateral, lateral, ventral and ventrolateral directions (see Fig. 11a for an example).

### Comparing morphological properties of XII MNs between embryonic day 17–18 and postnatal day 0

**Fig. 3:**
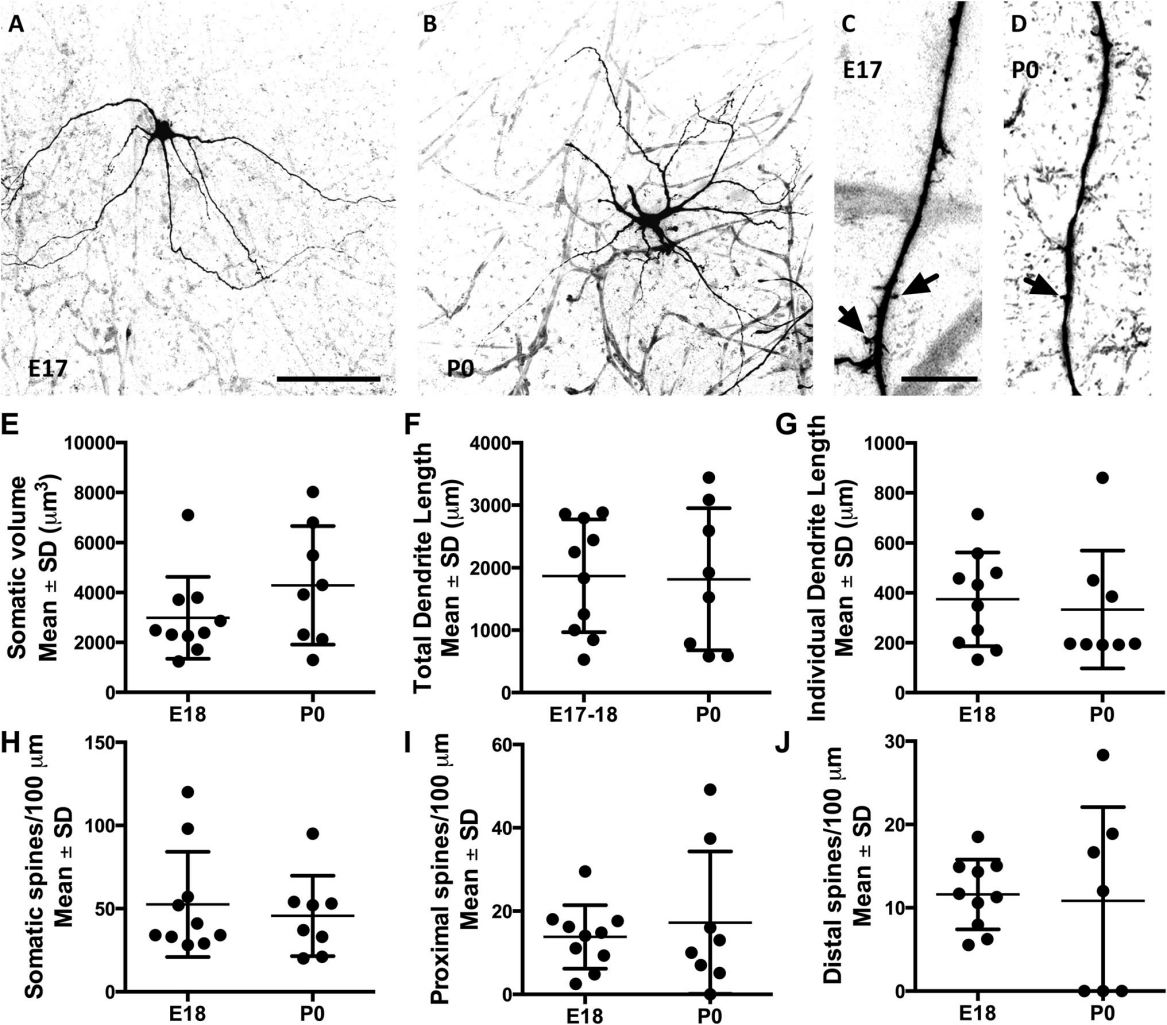
No significant morphological differences were found between E17–E18 and P0 XII MNs. **a**, **b** The branching morphology of an E17 and a P0 XII MN at low magnification, respectively. **c**, **d** The morphology of typical distal dendrites, including spines (indicated by *arrows*), of an E17 and P0 XII MN at high magnification, respectively. **e**–**j** Scatter plots of morphological measurements (somatic volume, total dendritic length, individual dendritic tree length, somatic spines, proximal spines and distal spines) from all E17–E18 and P0 XII MNs, with the mean measurement for each age group and standard deviation (SD) superimposed; no measurement showed a significant difference between E17 and E18 (*n* = 10) and P0 (*n* = 8) XII MNs; unpaired two-tailed Students’ *t* test, *P* > 0.05. *Scale bars* **a** and **b** 100 µm, **c** 5 µm

We initially compared the morphological properties of XII MNs immediately before birth (E17–18; *n* = 10) and soon after birth (P0; *n* = 8) groups. We found no significant difference between the two groups when we compared their cell volume, total dendritic length, the length of individual dendritic trees, or the number of spines on the soma, proximal and distal dendrites (Fig. 3). The cell volume was 2988 ± 1645 (mean ± SD for all measurements) μm^3^ at E17–18 and 4284 ± 2370 μm^3^ at P0; this change was not significant (Fig. 3e, unpaired *t* test, *P* = 0.19, *t* = 1.370, *df* = 16). The total dendritic length was 1869 ± 901 μm at E17–18 compared to 1815 ± 1137 μm at P0 (Fig. 3f, unpaired *t* test, *P* = 0.91, *t* = 0.1127, *df* = 16). The length of individual dendritic trees was 375 ± 188 μm at E17–18 and 333 ± 236 μm at P0 (Fig. 3g, unpaired *t* test, *P* = 0.68, *t* = 0.4140, *df* = 16). The number of spines located on the somata was 53 ± 32 at E17–18 and 46 ± 24 at P0 (Fig. 3h, unpaired *t* test, *P* = 0.52, *t* = 0.5140, *df* = 16). The number of spines located on the proximal and distal dendrites was 14 ± 8 and 12 ± 17 at E17–18, respectively, and 12 ± 4 and 11 ± 11 at P0, respectively (Fig. 3i, j, unpaired *t* test, *P* = 0.58, *t* = 0.5671, *df* = 16, and 0.84, *t* = 0.1974, *df* = 15). As there were no significant differences in morphology of XII MNs between the two age groups, we have grouped these results together as E17–P0.

### Changes in XII MN morphology during development from E17 to P28

The transition from the in utero environment to postnatal air breathing and suckling at P0 imposes a major change in function for XII MNs (Greer and Funk 2005), while the transition to a mature respiratory rhythm and dry food consumption occurs at 14–15 days post-birth (Kubin and Volgin 2008; Paton and Richter 1995). We therefore arbitrarily divided the MNs into 5 major age groups, corresponding to before (<P1) and after (>P13) these two major developmental transitions, and 3 approximately equal periods between P1 and P13: E17–P0 (*n* = 18), P1–4 (*n* = 27), P5–8 (*n* = 20), P9–13 (*n* = 16) and P14–28 (*n* = 22). We compared the total dendritic length, the length and diameter of individual dendritic trees, the length of major and minor cell axes, soma surface area and volume, and the number of spines on the soma, proximal and distal dendrites among these age groups. Results for each measurement will be presented in separate sections.

#### Changes in soma structure and volume during development

**Fig. 4:**
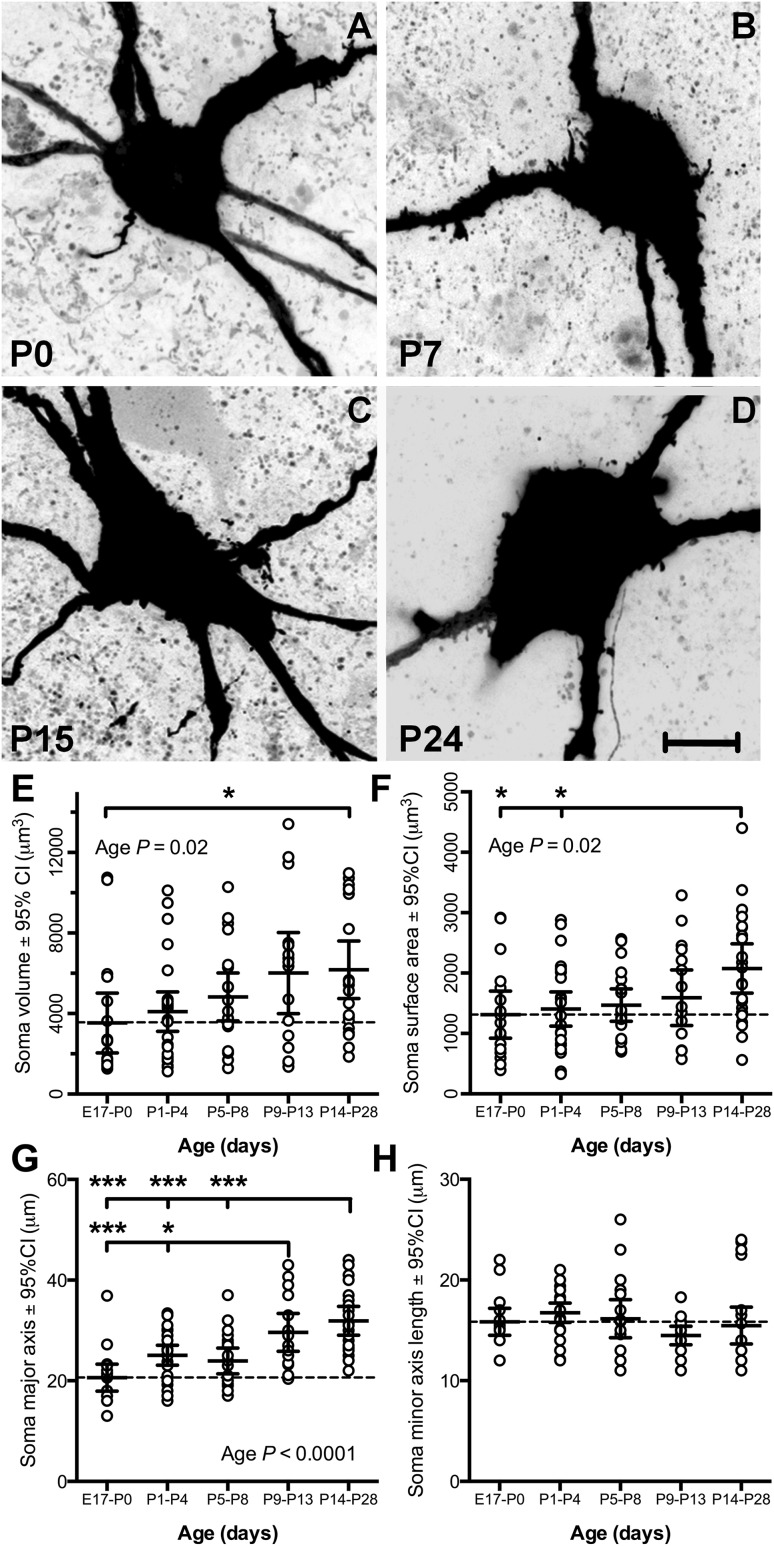
Developmental changes in XII MN somata between E17 and P28. **a**–**d** High magnification images of the cell soma and proximal dendrites of typical XII MNs from mice aged P0, P7, P15 and P24, respectively. **e**–**h** Scatterplots of morphological measurements (somatic volume, somatic surface area, major and minor somatic axis length) from all E17–P0, P1–P4, P5–P8, P9–P13 and P14–P28 XII MNs, with the mean measurement for each age group and 95 % confidence interval (CI) superimposed; a *dashed line* shows the mean parameter value at birth. Significant increases with increasing age were seen for somatic volume and surface area, and major somatic axis length, but not for minor somatic axis length; one-way ANOVA with age group variable, with Tukey’s multiple comparison between all age groups (*P < 0.05, **P < 0.01, ***P < 0.001). *Scale bar* 10 µm

The cell bodies of filled XII MNs were ellipsoid in shape (Fig. 4a–d), with their major axis oriented in the transverse plane. The cell body volume gradually and consistently increased with increasing age (e.g., compare Fig. 4a with d). By comparison to soma volume at E17–P0, P1–4 and P5–8 mean cell volume was 14 and 35 % greater, respectively, reaching a relatively stable increase of 69 % at P9–13 with only a slight further increase to 72 % at P14–28 (see mean values in Table 1, and cell values in Fig. 4e). Comparison of mean cell volume between age groups found a significant effect of age [*P* = 0.02, one-way ANOVA for age group, *F* (*dfn* = 4, *dfd* = 98) = 2.925], with the E17–P0 age group showing a significant difference to the P14–28 age group (*P* = 0.047, Tukey’s multiple comparison post-test, *q* = 3.961, *df* = 98). Cell body surface area was also increased during development [see mean values given in Table 1 and cell values in Fig. 4f, *P* = 0.02, one-way ANOVA for age group, *F* (4, 98) = 3.027], with the P14–28 age group showing a significant increase of 58 %, compared to the E17–P0 age group (*P* = 0.02, Tukey’s multiple comparison post-test, *q* = 4.373, *df* = 98; Fig. 4f) and P1–4 age group (*P* = 0.03, Tukey’s multiple comparison post-test, *q* = 4.243, *df* = 98; Fig. 4f).

**Table 1:** XII MN soma morphology measurements

Age group (days)	Volume*, ^^^^^ (μm^3^)	Surface area*, ^^^^ (μm^2^)	Major axis length****, ^^^^^^ (μm)	Minor axis length (μm)	Number of XII MNs
E17–P0	3531 ± 2992	1312 ± 786	20.6 ± 5.4	15.9 ± 2.7	18
P1–P4	4095 ± 2470	1387 ± 716	25.1 ± 5	16.8 ± 2.4	27
P5–P8	4824 ± 2537	1578 ± 574	23.9 ± 5.4	16.2 ± 4	20
P9–P13	6013 ± 3786	1603 ± 863	29.6 ± 7 ***E17–P0 *P1–4	14.5 ± 1.7	16
P14–P28	6124 ± 3219 *E17–P0	2076 ± 922 *E17–P0	31.9 ± 6.4 ****E17–P0, ***P1–4, P5–8	15.5 ± 4.1	22

Mean ± SD. All measurements were made from the same set of XII MN somata for each age group. * *P* < 0.05, ** *P* < 0.01, *** *P* < 0.001, **** *P* < 0.0001, one-way ANOVA for age group with Tukey’s multiple comparison post-test; ^^^ *P* < 0.05, ^^^^ *P* < 0.01, ^^^^^ *P* < 0.001, ^^^^ *P* < 0.0001, test for linear trend against age group

In order to directly compare our data to previous measurements of MN somata (Ulfhake and Cullheim 1988; Núñez-Abades and Cameron 1995), we also measured the length of the major and minor axes of the soma. The length of the major axis of the cell body increased significantly with age [Fig. 4g, *P* < 0.0001, one-way ANOVA for age group, *F* (4, 98) = 11.73], while the minor axis length was not significantly altered [Fig. 4h, *P* = 0.24, *F* (4, 98) = 1.391; see mean axis length values given in Table 1]. For the major axis length, the P14–28 age group was significantly longer than for each of the 3 youngest age groups (*P* < 0.001 for each comparison, Tukey’s multiple comparison post-test, *q* > 5.766 and *df* = 98 for all comparisons; Fig. 4g, upper statistical bar). The major axis length of the P9–13 age group was also significantly longer than the E17–P0 (*P* = 0.0002, *q* = 6.34 and *df* = 98) and P1–4 (*P* = 0.04, *q* = 4.104 and *df* = 98) age groups (Fig. 4g, lower statistical bar; Tukey’s multiple comparison post-test).

As the cell volume, surface and major axis length all increased significantly with age, we estimated the effect of age on each of these measurements. Given that three age groups of 4 days each separate the youngest and oldest age groups, we treated the 5 age groups as equally spaced time points (*x* variable) and fitted the mean data by linear regression. The linear trend for cell body volume was highly significant (*P* = 0.003, *F* (1, 3) = 79.7, *r*^2^ = 0.96, slope = 721 μm^3^ per age group). Similar tests for linear trend against age group for cell surface area [*P* = 0.035, *F* (1, 3) = 13.4, *r*^2^ = 0.82, slope = 172 μm^2^ per age group] and major axis lengths [*P* < 0.01, *F* (1, 3) = 26.8, *r*^2^ = 0.9, slope = 3 μm per age group] were significant, while there was no significant linear trend for minor axis length [*P* = 0.32, *F* (1, 3) = 1.4].

#### Changes in dendritic branching, length and diameter during development

**Fig. 5:**
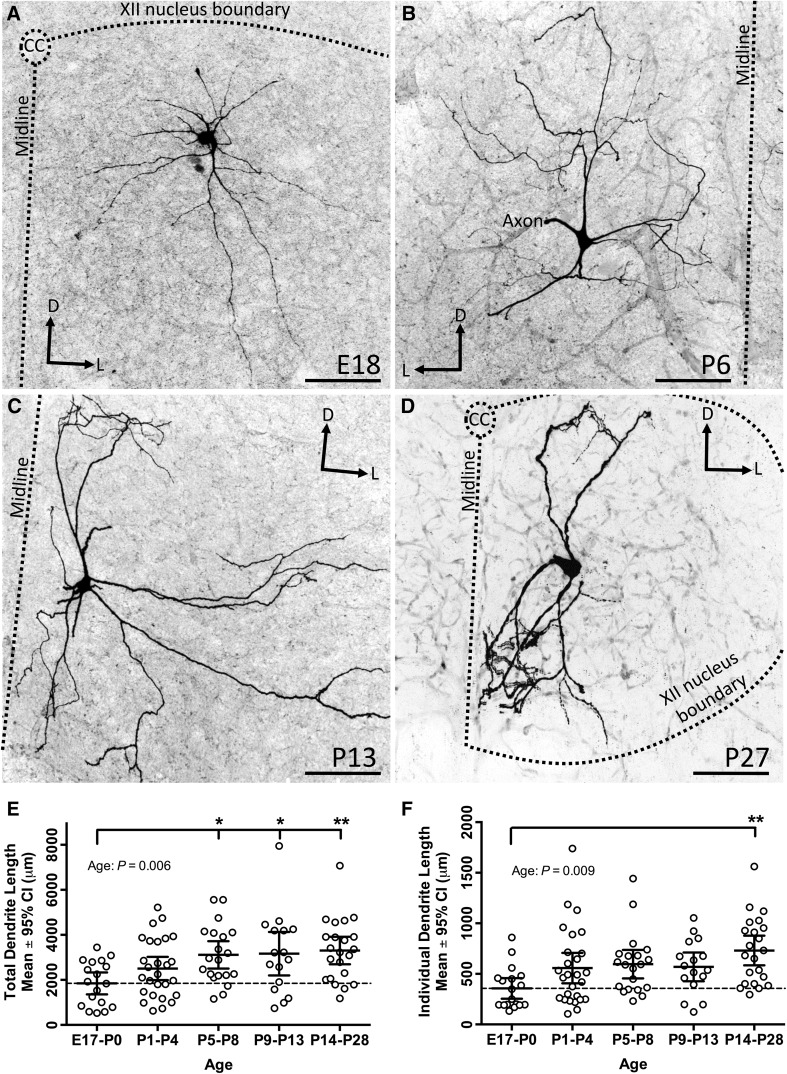
Dendritic length increases during postnatal development. **a**–**d** Low power images of the typical dendritic branching of XII MNs from mice aged E18, P6, P13 and P27, respectively; dorsal (*D*) and lateral (*L*) directions are indicated by *arrows*, the *midline* is indicated by a *dashed straight line* (in **a**–**d**), the central canal (*CC*) by a *dashed circle* (in **a**, **d**) and the boundary of the XII motor nucleus by a *dashed line* (in **a**, **d**), when they fell within the image boundary. **e**, **f** Scatterplots of dendritic measurements (total dendrite length per cell, and length of individual dendrites) from all E17–P0, P1–P4, P5–8, P9–P13 and P14–P28 XII MNs, with the mean measurement for each age group and 95 % confidence interval (CI) superimposed; a *dashed line* shows the mean parameter value at birth. A significant increase in total dendritic length was present between E17–P0 and P5–P8, P9–P13 and P14–P28 age groups, and in individual dendrite length between E17–P0 and P14–P28 age groups. One-way ANOVA with age group variable, with Tukey’s multiple comparison post-test. *Scale bars* for **a**–**d** 100 μm; *P < 0.05 and **P < 0.01

Representative examples of the dendritic branching of XII MNs from 4 different age groups are shown in Fig. 5a–d, illustrating the multipolar branching patterns seen, with confinement of the dendritic tree within the XII motor nucleus. Total dendrite length of the entire XII MN dendritic tree significantly increased over the 4 weeks of postnatal development [Fig. 5e; Table 2, one-way ANOVA with age group as variable, *P* = 0.006, *F* (4, 98) = 3.84]. Mean total dendrite length was approximately 1800 μm at E17–P0 (Fig. 5e). Using the E17–P0 length as a baseline (dashed line in Fig. 5e), total dendrite length had increased by 37 % at P1–P4 and by 69 % at P5–P8, then remained relative stable (71 %) at P9–P13, with a further small increase to 79 % at P14–P28 (Table 2; Fig. 5e). The age-dependent increase in total dendrite length was significant (Tukey’s multiple comparison post-test to E17–P0) for the P–58, P9–13, and P14–28 age groups, *P* < 0.05, *q* > 4 and *df* = 98 for all). A post-test for linear trend by age group was highly significant [*P* = 0.002, *F* (1, 3) = 19.1, *r*^2^ = 0.86, slope = 358 μm per age group].

**Table 2:** Measurements of XII MN dendrite length, surface area and volume

Age group (days)	Total dendrite length (μm)	Individual dendrite length (μm)	Total dendrite volume (μm^3^)	Total dendrite surface area (μm^2^)	Number of XII MNs
E17–P0	1845 ± 981	356 ± 205	796,806 ± 1.374^e+6^	105,992 ± 94,310	18
P1–P4	2506 ± 1303	557 ± 380	419,931 ± 462,670	89,404 ± 64,384	27
P5–P8	3111 ± 1295	596 ± 300	567,940 ± 474,495	104,964 ± 59,186	20
P9–P13	3164 ± 1812	569 ± 266	522,007 ± 351,174	117,977 ± 63,508	16
P14–P28	3305 ± 1373	731 ± 331	505,471 ± 463,078	109,531 ± 58,090	22
*P* (age group)	0.006	0.009	0.52	0.86	
*P* (post-test)	E17–P0 vs	E17–P0 vs			
	*P5–P8	**P14–P28			
	*P9–P13	^^^			
	**P14–P28 ^^^				

Mean ± SD. All measurements were made from the same set of XII MNs for each age group. * P < 0.05, ** P < 0.01, one-way ANOVA for age group with Tukey’s multiple comparison post-test; ^ *P* < 0.05, test for linear trend against age group

A significant developmental increase was seen in the length of individual dendritic trees (Fig. 5f; Table 2, one-way ANOVA with age group as variable, *P* = 0.009). The mean individual dendritic tree length was approximately 350 μm at E17–P0, increased in length by 57 % at P1–P4, was relatively stable at P5–P8 (67 %) and P9–P13 (55 %), and then increased significantly by 105 % of length at E17–P0 by P14–P28 (Tukey’s multiple comparison post-test to E17–P0, *P* = 0.003, *q* = 5.34, *df* = 97). Despite the period of mild individual dendrite retraction at P9–P13, a post-test for linear trend by age group was still significant [*P* = 0.04, *F* (1, 3) = 12.23, *r*^2^ = 0.80, slope = 76 μm per age group]. Despite these significant age-related increases in total and individual dendrite length, the total volume and surface area of the dendritic tree remained relatively stable during development [Table 2; one-way ANOVA with age group as variable, *P* = 0.52, *F* (4, 98) = 0.82 and *P* = 0.72, *F* (4, 98) = 0.52, respectively].

**Fig. 6:**
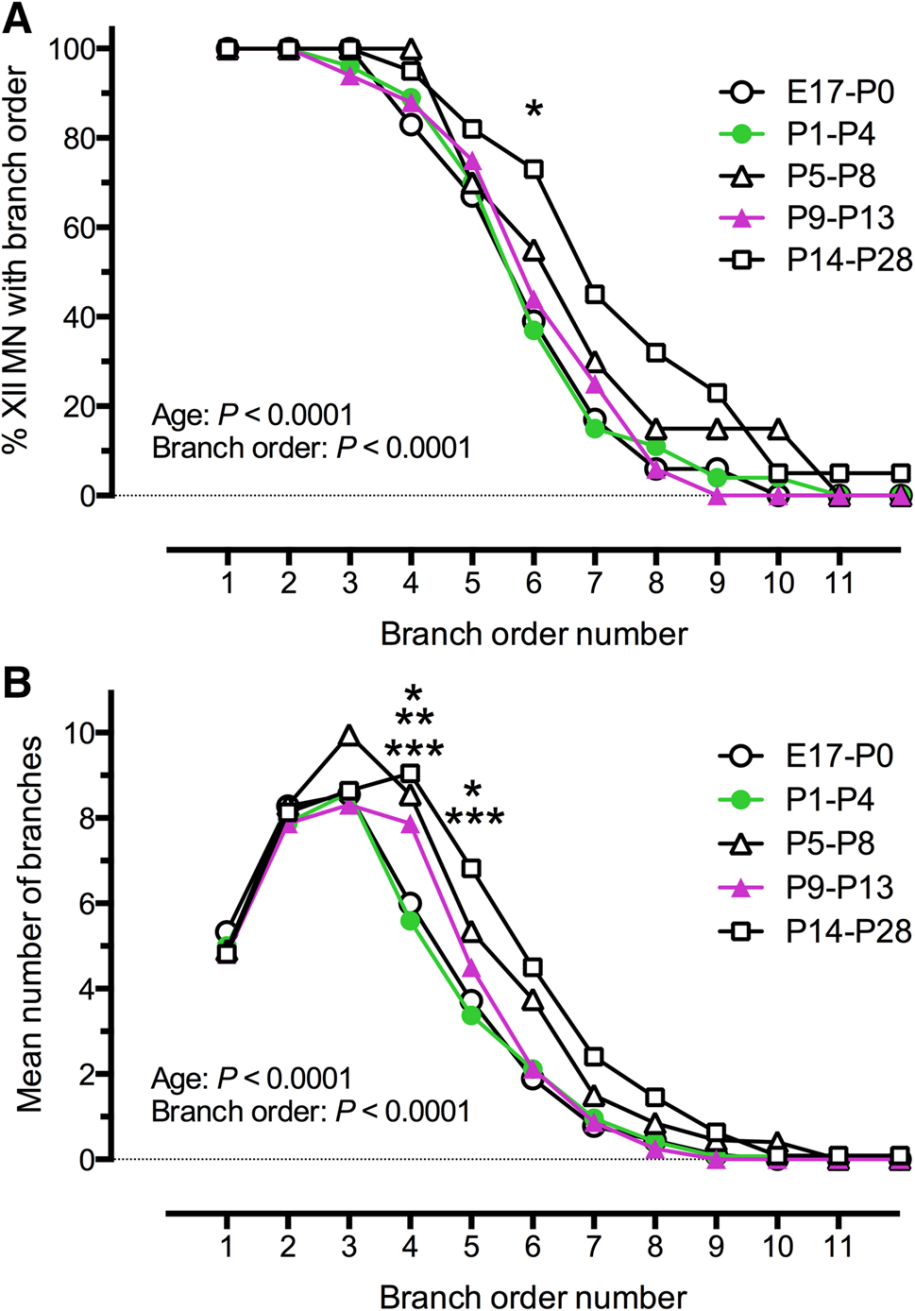
The % of XII MNs with higher dendritic branch orders and the number of dendrite branches per branch order increased with age. **a** The % of XII MNs with increasing levels of dendrite branch order; all MNs at all ages had 1st and 2nd order dendrites, while 3rd and 4th order dendrites were present in all P14–P28 MNs, which also showed a significantly higher percentage of MNs with 6th order dendrites, compared to E17–P0 MNs (two-way ANOVA with variables of age group and branch order number, Tukey’s multiple comparison test, *P* < 0.05 for E17–P0). **b** The mean number of dendrites for each branch order in each age group studied. The number of dendrites increased up to 3rd order branches and then declined with increasing branch order and MNs from the P14–P28 age group had more dendrites in the 4th and 5th branch orders, compared with both E17–P0 and P1–P4, and P5–P8 MNs had more 4th order branches compared to P1–P4 MNs (two-way ANOVA with variables of age group and branch order number, Tukey’s multiple comparison test. *P < 0.05, **P < 0.01, ***P < 0.001)

Next, we counted the percentage of XII MNs that had different branch orders (Table 3; Fig. 6a) and the number of dendritic branches per branch order (Table 3; Fig. 6b). In each age group, all filled XII MNs had 1st and 2nd order branches, 3rd order branches were present in all age groups but P9–13, and there was a progressive decline in the number of MNs with successively higher order branches in all age groups. A two-way ANOVA with age (*P* < 0.0001, *F* = 10, *df* = 4) and branch order as variables (*P* < 0.0001, *F* = 269, *df* = 11) showed a significant increase in the  % of P14–P28 MNs with 6th order branches, compared to P1–P4 XII MNs (*P* = 0.048, *F* = 6.2, *df* = 44; Tukey’s multiple comparison). The number of branches for higher branch order (Fig. 6b; Table 3) was also dependent on both developmental age (*P* < 0.0001, *F* = 6.3, *df* = 4) and branch order (*P* < 0.0001, *F* = 124, *df* = 11, two-way ANOVA with age group and branch order as variables). There was a significant increase in the number of 4th order branches at P14–P28, compared to both E17–P0 (*P* = 0.02, *q* = 4.4, *df* = 1176) and to P1–P4 (*P* < 0.001, *q* = 5.7, *df* = 1176), and for P1–P4, compared to P5–P8 (*P* = 0.009, *q* = 4.6, *df* = 1176), Tukey’s multiple comparison). There was also a significant increase in the number of 5th order branches at P14–P28, compared to both E17–P0 (*P* = 0.01, *q* = 4.5, *df* = 1176) and to P1–P4 (*P* = 0.0008, *q* = 5.6, *df* = 1176). These results show that the percentage of XII MNs with higher order branches and the number of branches per branch order only changed significantly at the 4th to 6th branch orders during postnatal development, by contrast to relatively stable dendrite branching patterns for lower branch orders.

**Table 3:** The percentage of XII MNs with branches of each branch order, and the mean number of branches for each branch order

Branch order	Number of MNs with branch order (%) Number of branches per branch order				
	E17–P0	P1–P4	P5–P8	P9–P13	P14–P28
1	18 (100 %) 5.3 ± 1.9	27 (100 %) 5 ± 1.5	20 (100 %) 4.9 ± 1.6	16 (100 %) 4.8 ± 1.5	22 (100 %) 4.8 ± 1.3
2	18 (100 %) 8.3 ± 3.4	27 (100 %) 7.8 ± 2.5	20 (100 %) 8.1 ± 2.4	16 (100 %) 7.9 ± 2.5	22 (100 %) 8.2 ± 2.5
3	18 (100 %) 8.6 ± 5.4	26 (96 %) 8.9 ± 4.1	20 (100 %) 10.1 ± 3.7	15 (94 %) 8.3 ± 4.8	22 (100 %) 8.4 ± 3.5
4	17 (94 %) 6 ± 5.9*	24 (89 %) 6.1 ± 3.9***	20 (100 %) 8.8 ± 5.4*	14 (88 %) 7.9 ± 6.9	21 (95 %) 8.2 ± 5.1
5	13 (72 %) 3.7 ± 4.1*	19 (70 %) 3.7 ± 3.5***	14 (70 %) 5.8 ± 6.0	11 (69 %) 4.5 ± 4.4	17 (77 %) 5.9 ± 6.2
6	8 (44 %) 1.9 ± 3.1	10 (37 %)* 2.6 ± 3.7	11 (55 %) 4.2 ± 5.3	5 (31 %) 2.1 ± 3	15 (68 %) 4.3 ± 5.4
7	3 (17 %) 0.8 ± 2.1	6 (22 %) 1.4 ± 2.7	6 (30 %) 1.7 ± 2.6	3 (19 %) 0.9 ± 2.1	10 (45 %) 2.7 ± 4
8	1 (6 %) 0.4 ± 1.9	3 (11 %) 0.6 ± 1.4	3 (15 %) 0.9 ± 2.1	1 (6 %) 0.3 ± 1	6 (27 %) 1.6 ± 2.6
9	1 (6 %) 0.1 ± 0.5	1 (4 %) 0.1 ± 0.4	3 (15 %) 0.5 ± 1.1	0 (0 %)	4 (18 %) 0.6 ± 1.3
*P* (age) **** ****	*vs P14–P28	***vs P14–P28 *vs P5–P8	*vs P1–P4		
*P* (branch) **** ****					

Mean ± SD. * P < 0.05, ** P < 0.01, *** P < 0.001, **** P < 0.0001 for two-way ANOVA with variables of age group and branch order, with Tukey’s multiple comparison post-test

**Fig. 7:**
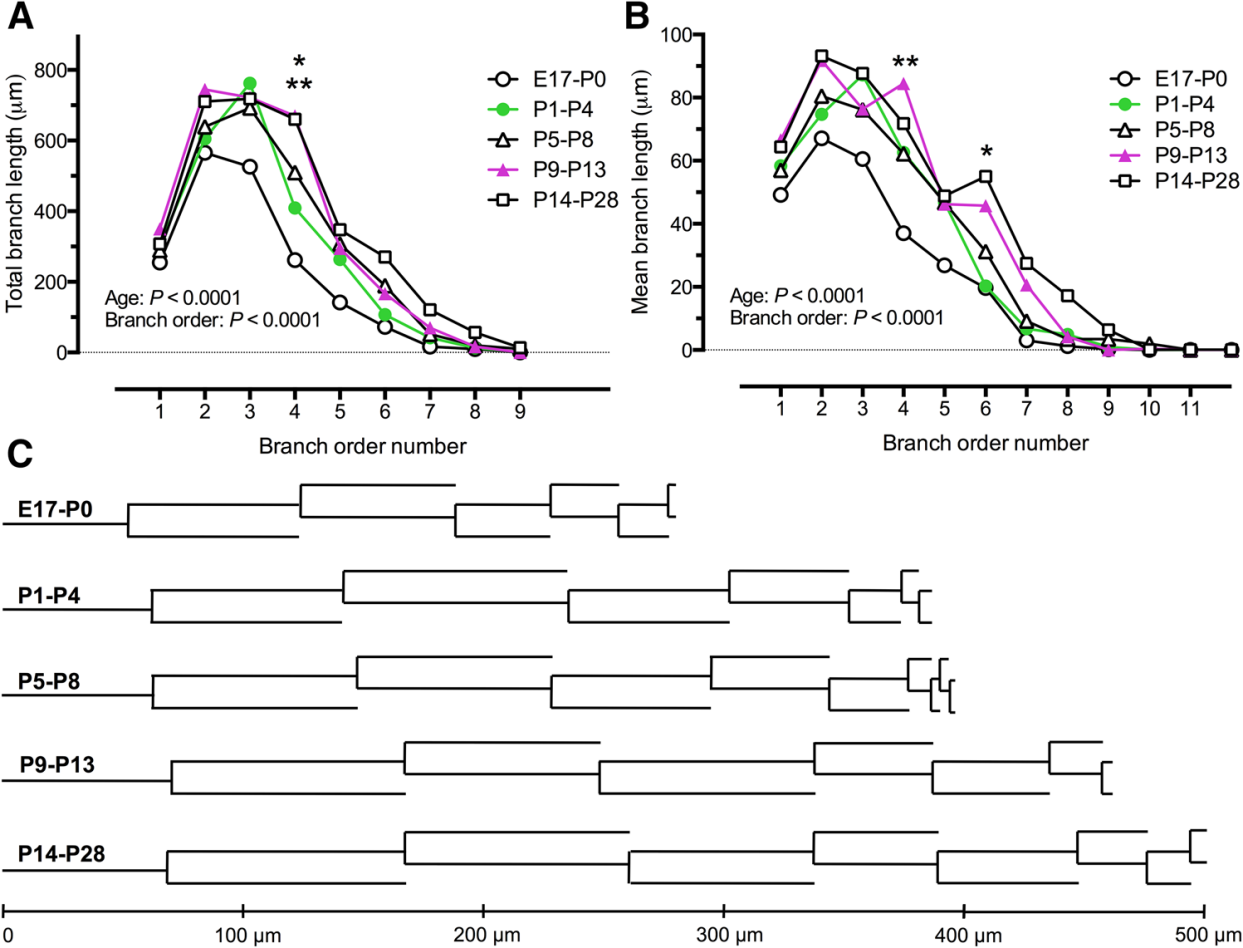
The total and individual branch order lengths of XII MNs increased with age. **a** Mean total branch length for each branch order in each age group, which significantly increased with age; the total length of 4th order branches was significantly greater at P9–P13 (**P < 0.01) and P14–P28 (***P < 0.001), compared to E17–P0. **b** The mean branch length of individual dendrites, which also increased with age; the length of 4th branch order dendrites increased (E17–E18 compared with P9–P13, ***P < 0.01, as did the length of 6th branch order dendrites (E17–E18 compared with P14–P28, *P < 0.05). Two-way ANOVA with variables of age group and branch order number, Tukey’s multiple comparison post-test for both data sets. **c** Dendrograms constructed from the mean branch lengths for XII MNs in each age group, for all branch orders that had at least 3 MNs with dendrites of that branch order

We measured the total branch length and mean length of dendrites for each branch order (Table 4; Fig. 7a, b, respectively). The 1st order total branch length was relatively stable, while total branch length for 2nd order branches increased sharply for all ages, then remained relatively constant for 3rd order branches, with higher order branches showing decreasing total branch length [Fig. 7a; Table 4, two-way ANOVA with age group (*F* = 7.4, *df* = 4) and branch order (*F* = 86, *df* = 8) as variables, *P* < 0.0001 for both]. Total branch length for 4th order branches was significantly increased at P9–P13 (Fig. 7a, filled triangles) and P14–P28 (Fig. 7a, open squares) age groups, compared to the E17–P0 (Fig. 7a, open circles) age group (*P* < 0.05, *q* > 5.9, *df* = 882, Tukey’s multiple comparison for both). The mean branch length changed in a similar fashion to total branch length, during development [Fig. 7b; Table 4, two-way ANOVA with age group (*F* = 12, *df* = 4) and branch order (*F* = 122, *df* = 11) as variables, *P* < 0.0001 for both]. For the 4th branch order, there was a significant increase in mean branch length from E17 to P0 (open circles) to P9–P13 (filled triangles, 4th order branch mean length *P* = 0.003, *q* = 6.7, *df* = 1176), Tukey’s multiple comparison). The mean branch length of 6th order branches also increased significantly from P1–P4 to P14–P28 (*P* = 0.04, *q* = 5.9, *df* = 1176).

**Table 4:** XII MN single dendrite diameter and equivalent cylinder diameter for 1st, 2nd, 3rd and terminal branches

Branch order	Single dendrite and equivalent cylinder diameters				
	E17–P0	P1–P4	P5–P8	P9–P13	P14–P28
1	4.2 ± 0.7 22.4 ± 9.7	4 ± 0.8 20.1 ± 8	4.5 ± 0.7 21.7 ± 7.6	4.3 ± 0.7 19.5 ± 6.6	4.1 ± 0.6 20 ± 6.5
2	2.1 ± 0.8 17.1 ± 8.1	1.8 ± 0.5 13.9 ± 6.6	2.4 ± 0.6* 20.2 ± 9.4	2.3 ± 0.7 17 ± 8	2.3 ± 0.7 19.3 ± 8.2
3	1.2 ± 0.5 10.2 ± 8.7	1.2 ± 0.4 10.3 ± 7	1.3 ± 0.5 12.6 ± 7.2	1.6 ± 0.2 13.9 ± 8.9	1.6 ± 0.6 14.6 ± 7.3
Terminal	0.9 ± 0.4 4.8 ± 4.3	0.8 ± 0.3 4.6 ± 3.8	0.9 ± 0.4 6.7 ± 3.4	0.8 ± 0.2 6.5 ± 6.2	0.9 ± 0.4 6.7 ± 4
*P* (age)	***		*vs P1–P4		
*P* (branch)	**** ****				

Mean ± SD. * P < 0.05, ** P < 0.01, *** P < 0.001, **** *P* < 0.0001 for two-way ANOVA with variables of age group and branch order, with Tukey’s multiple comparison post-test

Figure 7c summarizes developmental changes in mean branch length per branch order for 1st and higher (up to 10th) order branches as a dendrogram for each age group. These results show that, while the mean dendrite length for a given branch order generally increases with developmental age, significant changes in the branch length per branch order occur primarily at the 4th and 6th branch orders during postnatal development.

**Fig. 8:**
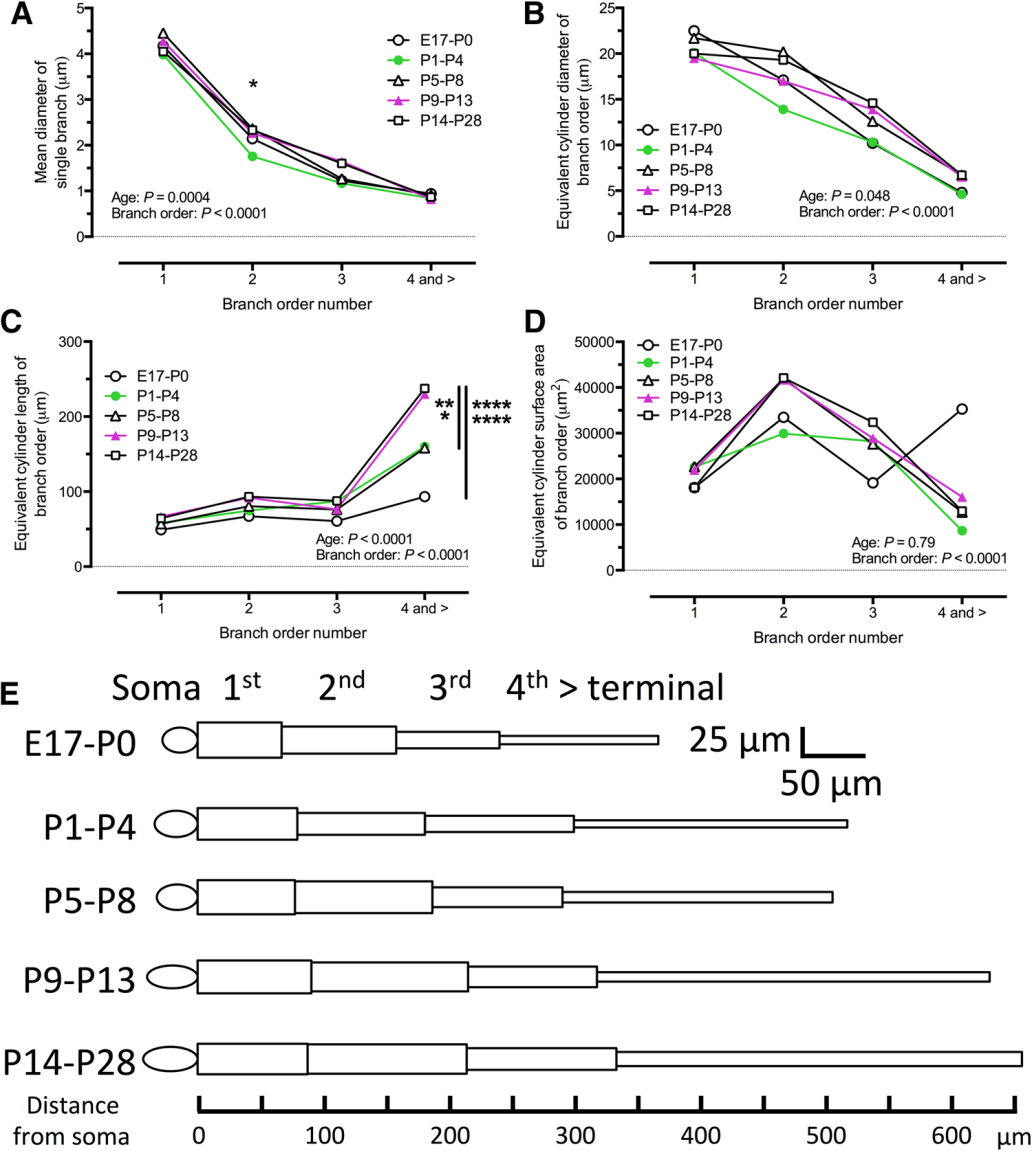
Equivalent cylinder parameter measurements for 1st to 3rd order branches and a terminal branch (sum of all 4th and higher order branches). **a** The mean diameter of all single branches for the 1st, 2nd, 3rd and terminal branches, for all age groups studied; branch diameter varied significantly with age and branch order, and the mean diameter of 2nd order branches was significantly increased for P5–P8, compared to P1–P4 XII MNs. **b** The equivalent cylinder diameter (being the single branch diameter × the number of branches for a given branch order) for the same branch orders as **a**, for all age groups; equivalent cylinder diameter varied significantly with both age group and branch order. **c** The equivalent cylinder length for the same branch orders as **a**, for all age groups; equivalent cylinder length varied significantly with both age group and branch order, with significant increases in the length of the equivalent cylinder for the terminal branch from E17–P0 to P9–P13 and P14–P28 (****P < 0.0001 for both), from P1–P4 to P14–P28 (**P < 0.01), and from P5–P8 to P14–P28 (*P < 0.05). **d** The equivalent cylinder surface area for the same branch orders as **a**; surface area varied significantly with branch order but not age group. Two-way ANOVA with age group and branch order as variables, Tukey’s multiple comparison post-test for all measurements. **e** The mean developmental changes in XII MN soma major and minor axes (represented as *ellipses*), and of 1st, 2nd, 3rd, and terminal branches, proportionally represented as single equivalent cylinders, for the 5 age groups analyzed in this study

The diameter, length and surface area of dendrites plays a critical role in determining the passive electrotonic characteristics of neurons (Rall 1959). We measured the mean diameter of individual 1st, 2nd and 3rd order dendrites, as these branch orders were present in virtually all filled XII MNs. As progressively fewer MNs had higher order dendrites (Fig. 6a), the number of branches for 4th and higher branch orders progressively declined (Fig. 6b), and the mean diameter of 4th and higher order dendrites varied relatively little (data not shown), we calculated a single mean diameter for all 4th and higher order dendrites for each XII MN. The mean diameters of single dendrites for these branch orders are shown in Fig. 8a and Table 4. Single dendrite diameter was significantly influenced by both age and branch order (two-way ANOVA, age group and branch order as variables, *P* = 0.0004, *F* = 5.2, *df* = 4 for age group, *P* < 0.0001, *F* = 658, *df* = 3 for branch order). Primary dendrite diameter was approximately 4 μm at E17–P0 and was virtually unchanged throughout development (Fig. 8a, all age symbols). While there was a decrease in single dendrite diameter for 2nd order dendrites in all age groups, 2nd order dendrite diameter was significantly larger for the P5–P8 age group compared to the P1–P4 age group (Fig. 8a, filled circles compared to all other age symbols; *P* = 0.04, *q* = 5.1, *df* = 392, Tukey’s multiple comparison). Tertiary branches decreased further in diameter, and 4th and higher order branch diameter also decreased to approximately 1 μm for all age groups.

The passive electrical characteristics of signal propagation in a morphologically complex dendritic tree can be approximated by collapsing individual dendrites of a given branch order into a single equivalent cylinder (Rall 1959, 1977). We calculated the diameter of the equivalent cylinder for 1st, 2nd, 3rd and higher order dendrites (Fig. 8b; Table 4) by multiplying the mean single dendrite diameter for a given branch order by the number of dendrite branches for the same branch order in each filled MN. The equivalent cylinder diameter declined significantly with increasing branch order [Fig. 8a, two-way ANOVA for age group (*P* = 0.07, *F* = 2.9, *df* = 4) and branch order (*P* < 0.0001, *F* = 97, *df* = 3)]. This decrease in equivalent cylinder diameter was highly linear with increasing branch order in MNs from the E17–P0 (Fig. 8a, open circles) and P1–P4 (Fig. 8a, filled circles) age groups (linear regression *r*^2^ of 0.99 for both age groups), but a progressive increase in 2nd order equivalent cylinder diameter, relative to 1st order equivalent cylinder diameter, was apparent in older (Fig. 8a, open and filled triangles and open squares) age groups (linear regression *r*^2^ of 0.95, 0.96 and 0.90 for P5–P8, P9–P13 and P14–P28 age groups, respectively). We calculated the equivalent cylinder length as the mean branch length for 1st, 2nd and 3rd order branches (see Fig. 7b), as virtually all MNs had up to 3rd order branches, and calculated the 4th order to terminal branch equivalent cylinder length as equal to the sum of mean branch lengths for 4th to terminal branch orders divided by the number of XII MNs which had 4th and higher order branches. For comparison to equivalent cylinder diameter, the equivalent cylinder lengths for these dendrite branch orders are plotted in Fig. 8c and given in Table 5. Equivalent cylinder length was significantly increased [two-way ANOVA, variables of age group (*P* < 0.0001, *F* = 8.2, *df* = 4) and branch order (*P* < 0.0001, *F* = 64, *df* = 3)]; while 1st to 3rd order equivalent cylinder length was relatively stable, marked and significant differences in length were seen for the 4th to terminal branch order (E17–P0 vs P9–14 and P14–8, *P* < 0.0001, *q* > 8.3, *df* = 385 for both; P1–P4 vs P14–28, *P* = 0.009, *F* = 5.7, *df* = 385; P5–P8 vs P14–28, *P* = 0.01, *F* = 5.6, *df* = 385; Tukey’s multiple comparison). Finally, as the equivalent cylinder surface area is linearly related to the electrotonic length of a dendritic compartment, we calculated equivalent cylinder surface area for each branch order using the standard formula for cylinder surface area [surface area = 2(*π* × radius^2^) + (2 × *π* × radius) × length; Fig. 8d; Table 5]; cylinder surface area was relatively stable for branch orders 1–3, but decreased markedly for the terminal branch order. Cylinder surface area was significantly increased with branch order (*P* < 0.0001, *F* = 9.8, *df* = 3) but not with age group (*P* = 0.79, *F* = 0.4, *df* = 4). These changes in equivalent cylinder representation of 1st, 2nd, 3rd and higher order dendrites are shown graphically in Fig. 8e.

**Table 5:** Measured XII MN equivalent cylinder length, with calculated cylinder volume and surface area for 1st, 2nd, 3rd and higher order branches

Branch order	Equivalent cylinder length				
	Equivalent cylinder volume				
	Equivalent cylinder surface area				
	E17–P0	P1–P4	P5–P8	P9–P13	P14–P28
1	49.2 ± 34 5755 ± 1145 18,081 ± 15,257	58.4 ± 32.7 7194 ± 1545 22,601 ± 25,229	57 ± 31.1 7219 ± 1656 22,678 ± 23,264	66.6 ± 28.2 6985 ± 1176 21,945 ± 14,779	64.4 ± 32.4 5754 ± 709 18,077 ± 10,448
2	67.1 ± 37.9 10,660 ± 2609 33,491 ± 34,773	74.7 ± 37.1 9523 ± 1925 29,917 ± 31,417	80.5 ± 44.4 13,340 ± 2189 41,910 ± 30,757	91.8 ± 40 13,264 ± 2328 41,669 ± 29,257	93.2 ± 43 13,404 ± 2296 42,111 ± 33,838
3	60.5 ± 31.1 6087 ± 14,151 19,121 ± 18,861	87.2 ± 41.4 8978 ± 1521 28,205 ± 24,836	76.2 ± 39.7 8805 ± 1474 27,660 ± 20,716	76.3 ± 33.7 9204 ± 1800 28,915 ± 22,621	87.7 ± 34.4 10,315 ± 2225 32,407 ± 32,792
4 and >	93.2 ± 61 11,236 ± 6255 35,298 ± 83,373	160.1 ± 112** 14,887 ± 4360 8681 ± 10,149	158.2 ± 85.1* 24,329 ± 7008 12,715 ± 11,367	230 ± 151.4**** 9204 ± 1800 16,032 ± 21,710	237.5 ± 139.5**** 23,917 ± 4878 12,954 ± 11,560
*P* (age)	****	**vs P14–P28	*vs P14–P28	****vs E17–P0	****vs E17–P0
*P* (branch)	**** ****				

Mean ± SD. * P < 0.05, ** *P* < 0.01, *** P < 0.001, **** P < 0.0001 for two-way ANOVA with variables of age group and branch order, with Tukey’s multiple comparison post-test

#### Commissural crossing of XII MN dendrites

**Fig. 9:**
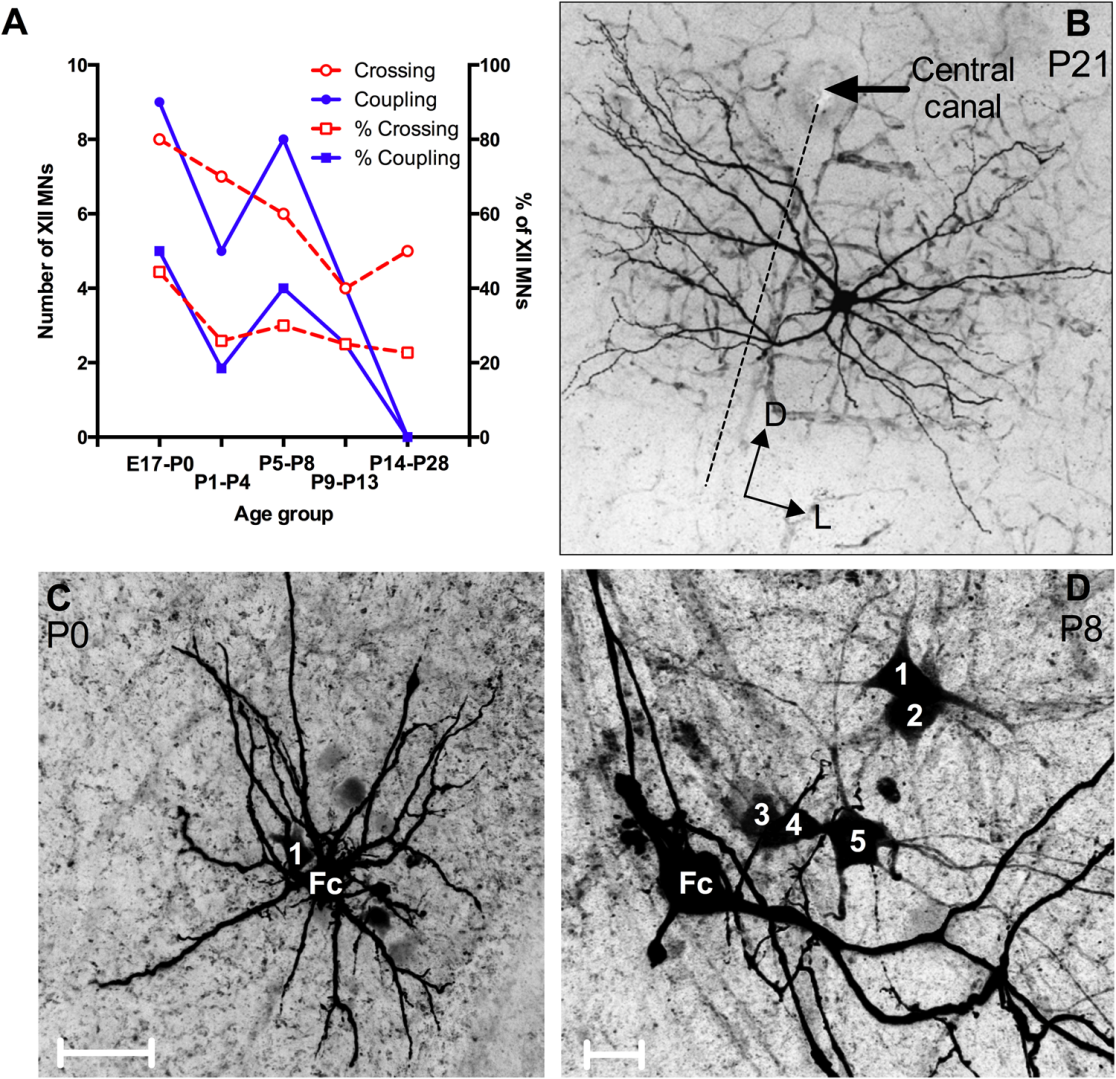
The presence of XII MN dendrites crossing the midline to the contralateral brainstem and dye-coupling between XII MNs decreases with developmental age. **a** The number of XII MNs (*left Y axis*) and the % of XII MNs with contralateral dendrites or dye-coupling in each age group. **b** An example of an XII MN from a P21 mouse, with dendrites crossing the midline (shown by *dashed line* and *arrow* indicating the central canal). **c** An example of soma–soma dye-coupling; it is the maximum intensity projection of a confocal *z*-stack with the somata of the Neurobiotin-filled cell (Fc) and of a single dye-coupled neuron (1) from a P0 mouse, with the two somata in close proximity. **d** An example of dendro-dendritic dye-coupling; it is the maximum intensity projection of a confocal *z*-stack with the somata of the Neurobiotin-filled cell (Fc) and of 5 dye-coupled neurons (1, 2, 3, 4, 5) from a P8 mouse, with dendrites as the only points of contact between the labeled cell and all dye-coupled MNs. *Scale bars* **c** 50 µm, **d** 20 µm

Thirty of 103 filled cells (29.1 %) from all age groups had medial and/or ventromedial dendrites that crossed the midline to the contralateral XII nucleus (Fig. 9a and example in Fig. 9b). Cells with contralaterally projecting dendrites are indicated by the white ‘×’ symbol superimposed on cell symbols in Fig. 2a, b. Of the 30 MNs with crossing dendrites, 27 of them (90 %) were located in the ventromedial subnucleus of the XII motor nucleus, while the remaining 3 (10 %) were located in the dorsal portion close to the border of the ventromedial subnucleus (Fig. 2a, b). Of the 64 MNs with somas located in the ventromedial subnucleus, 27 MNs (42.2 %) had dendrites crossing to the contralateral side. None of the 7 MNs located in the ventrolateral subnucleus had dendrites crossing the midline. There was a developmental decline in the number and the percentage of MNs that had dendrites crossing the midline (Fig. 9a). Dendrites of 8 of 18 filled MNs (44.4 %) from the E17–P0 age group showed a commissural crossing, compared to 7 of 27 MNs (25.9 %) from P1 to P4, 6 of 20 (30 %) MNs from P5 to P8, 4 of 16 MNs (25 %) from P9 to P13 group, and 5 of 22 MNs (22.7 %) from P14 to P28 age groups. There was also a rostro-caudal difference in the number of XII MNs with commissurally crossing dendrites (Fig. 2a); 3 of 15 (20 %) MNs in the rostral sector, 9 of 40 (22.5 %) and 10 of 30 (33.3 %) MNs in each of the rostral and caudal halves of the median sector, respectively, and 8 of 32 (25 %) MNs in the caudal sector had this morphological characteristic.

#### Dye-coupling between XII MNs

Neurobiotin can permeate between cells via connexon and pannexon ion channels present in gap junctions (Peinado et al. 1993; Chang et al. 1999; Kita and Armstrong 1991; Connors and Long 2004). The spread of Neurobiotin from a filled neuron to adjacent neurons thus indicates the presence of gap junctions, which allow electrotonic coupling between these neurons (Peinado et al. 1993; Chang et al. 1999; Kita and Armstrong 1991). Some filled XII MNs showed dye-coupling to neighboring XII MNs (Fig. 9c, d). XII MNs which were revealed by dye-coupling were distinguished by a fainter fill, which did not reliably extend into thin distal dendrites; we therefore did not include the morphology of any MNs revealed by dye-coupling in our main analysis.

The somata of dye-coupled cells were mostly within close proximity (<250 μm) of the filled XII MN and all dye-coupled cells were located in the same XII subnucleus as the filled XII MN, thus likely innervating the same muscle group. Dye-coupled MNs contacted either the soma (Fig. 9c) or the dendrites (Fig. 9d) of the Neurobiotin-filled XII MN. In approximately 80 % of cases, dye-coupling was clearly dendro-dendritic (Peinado et al. 1993), mostly via primary dendrites, and, in a few instances, via distal dendrites, as the filled XII MNs only came into close contact at these locations [Fig. 9d, the filled (indicated by ‘Lc’) neuron and its dye-coupled neurons 1–5)]. In the remaining ~20 % of the cases, soma-somatic or dendro-somatic close contacts were observed.

The prevalence of dye-coupling between XII MNs was age-dependent. In the E17–P0 age group, 9 of 18 MNs (50 %) displayed dye-coupling, compared to 5 of 27 MNs (18.5 %) at P1–P4, 8 of 20 (40 %) MNs at P5–P8, 4 of 16 (25 %) MNs at P9–P13, and 0 of 22 MNs at P14–P28 age groups (summarized in Fig. 9a). Dye-coupling was not limited to any particular XII region, but was distributed throughout the rostro-caudal extent of the hypoglossal motor nucleus, and was approximately equally distributed among the dorsal, ventromedial and ventrolateral subnuclei (see Fig. 2a). For all dye-coupled MNs (*n* = 25, ages between E17 and P13), dye-coupling was seen in 11 of 32 (34.4 %) MNs located in the dorsal subnucleus, 14 of 47 (29.8 %) MNs located in the ventromedial subnucleus, and 1 of 4 (25 %) MNs located in the ventrolateral subnucleus. For the same set of MNs, dye-coupling was seen in 8 of 24 (33.3 %) MNs from the caudal portion of the hypoglossal nucleus, 14 of 48 (29.2 %) MNs located in the median portion, and 4 of 11 (36.4 %) MNs located in the rostral portion.

All dye-coupled MNs were located ipsilateral to the XII MN filled with Neurobiotin. Although 8 MNs with dendrites crossing the midline also displayed dye-coupling to MNs located in the ipsilateral XII nucleus, no examples were observed of XII MNs with dye-coupling across the midline.

#### Spine distribution and density

**Fig. 10:**
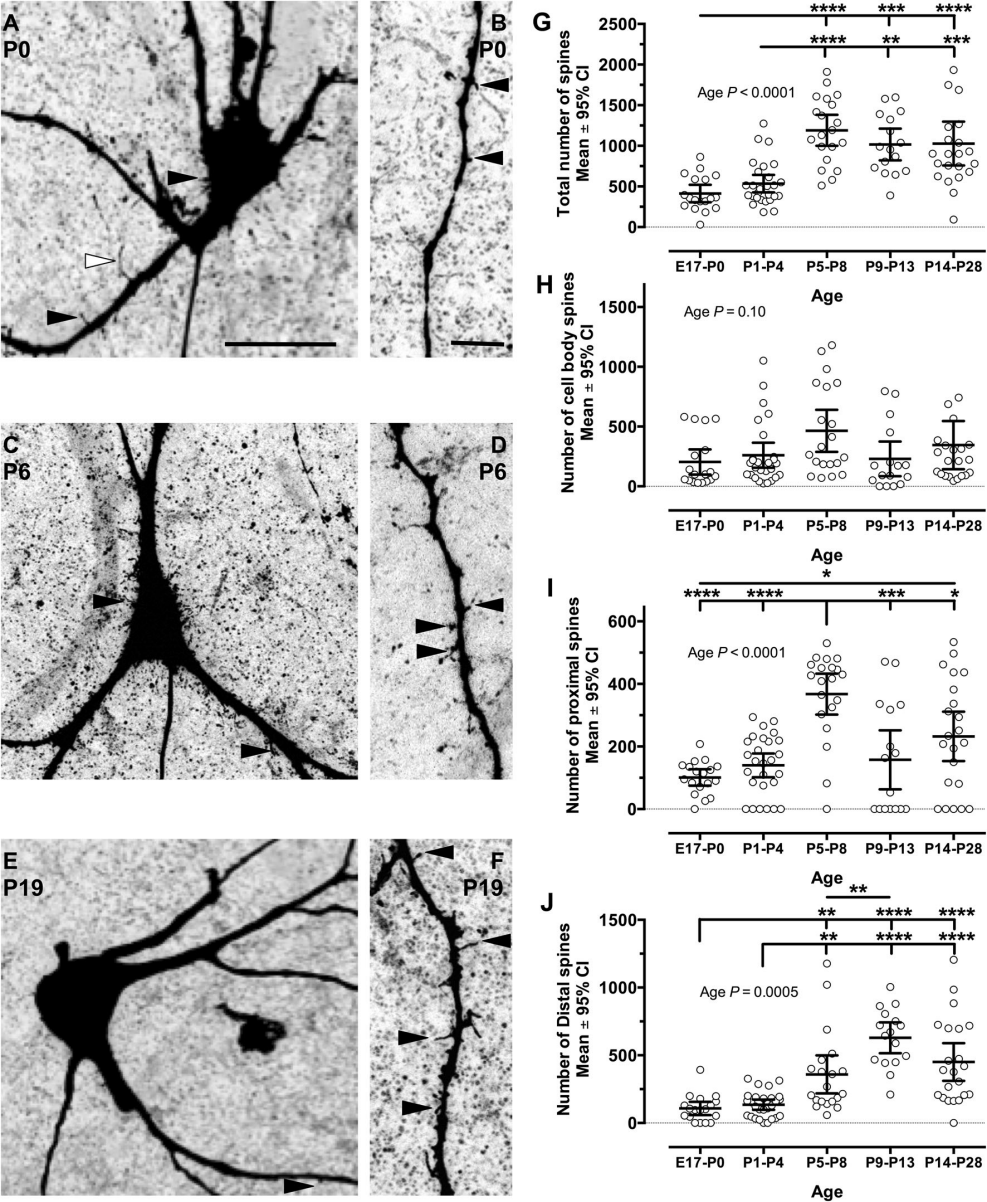
Spine number and distribution change through the postnatal developmental period. **a**, **c**, **e** Examples of somatic and proximal dendrite spines, at P0, P6 and P19, respectively. **b**, **d**, **f** Examples of distal dendrite spines at P0, P6 and P19, respectively. **g** The total number of spines present on the soma, proximal dendrites and distal dendrites of all XII MNs in this study; the number of spines increases throughout development to P5–P8 and then remains stable. Total spine number at P5–P8, P9–P13 and P14–P28 age groups was significantly larger than at either E17–P0 or P1–4 age groups. By contrast, the total number of spines present on the soma (**h**) was relatively stable throughout development. The number of spines on proximal dendrites (**i**) was increased significantly at P5–P8 compared to all other age groups, as well as at P14–P28, compared to E17–P0. **j** The total number of spines on distal dendrites, which increased significantly from both E17–P0 and P1–P4 to all of P5–P8, P9–P13 and P14–P28 groups, and was also significantly increased from P5–P8 to P9–P13. One-way ANOVA with Tukey’s post-test; *P < 0.05, **P < 0.01, ***P < 0.001 or ****P < 0.0001 for comparisons. *Scale bars* **a** 10 µm (applies to **c** and **e**), **b** 1 µm (applies to **d** and **f**)

In many neurons, dendritic spines are a prominent site of synaptic input and synaptic plasticity (Arellano et al. 2007; Alvarez and Sabatini 2007). A variety of small terminating processes (putative spines or filopodia; Gray 1959) were observed protruding from the somata and dendrites of XII MNs in all age groups (black arrow heads in Fig. 10a–f). Although dendritic spines are present on spinal (Cameron et al. 1985; Goshgarian and Rafols 1981) and cranial MNs (Yoshida et al. 1987; O’Kusky 1998), there is little quantitative information available about dendritic spine density and distribution in MNs. We have therefore quantitatively compared spine density on the XII MN cell body, primary (defined as 1st branch order) and distal dendrites (defined as 2nd and all higher branch orders) of XII MNs during development.

All clear protrusions from soma or dendritic shafts, of less than 5 μm in length, were counted as spines (Boyer et al. 1998), whereas longer processes were considered to be a terminal dendritic branch. Examples of structures counted as spines or filopodia are indicated by the black arrowheads in Fig. 10a–f, and an example of a short terminal branch example is indicated by a white arrowhead in Fig. 10a. We did not attempt to classify spines by shape, but note that all typical spine shapes (mushroom, stubby, thin and filopodia) were present (Harris and Weinberg 2012). For the cell body, the number of spines were directly counted as the number of protrusions from the perimeter of the cell body for each section in the confocal *z-*stack; we note that this method does potentially allow both double counting of spines appearing in successive sections or non-counting of spines straddling successive sections. For proximal and distal dendrites, protrusions were counted per 100 μm of dendrite length within a confocal section; over 65,000 μm of dendrite length, at all branch orders, were sampled to generate a spine density measure for each XII MN. The estimated total number of proximal and distal dendritic spines for each XII MN was then calculated as: (total dendritic length for a branch order/100 μm) × spine density per 100 μm for same branch order) (Routh et al. 2009).

The total number of spines per XII MN increased rapidly early in development until P5–P8 and then remained relatively constant (Fig. 10g). From an average of 412 spines at E17–P0, total spine number increased by 30 % at P1–P4 and a further 159 % at P5–P8, then declined slightly at P9–P13 and P14–P28; the mean total number of spines at P14–P28 was more than double (a 149 % increase) the mean total spine number at E17–P0. Total spine number was highly influenced by age (one-way ANOVA, variable age group, *P* < 0.0001, *F* = 14.9, *df* = 102); total spine counts at E17–P0 were significantly less than those at P5–P8, P9–P13 and P14–P28 (Tukey’s multiple comparison test, *P* < 0.001, *q* > 6.2, *df* = 98 for all), and counts at P1–P4 were significantly less than P5–P8, P9–P13 and P14–P28 counts (*P* < 0.01, *q* > 5.4, *df* = 98 for all).

While a similar pattern of increasing spine number to P5–P8, followed by a decline in spine number was seen, somatic spine number did not change significantly with age group (one-way ANOVA, variable age group, *P* = 0.10; Fig. 10h; Table 6), due to quite wide variation in somatic spine counts within each age group. As the soma surface area showed a progressive increase with age, we also calculated somatic spine density normalized by somatic surface area; spine density increased from E17–P0 to P5–P8, and then decreased to P14–P28 (Table 6). Somatic spine density also did not alter significantly with age group (one-way ANOVA, variable age group, *P* = 0.35, *F* = 1.1, *df* = 102; Fig. 10h; Table 6).

**Table 6:** Spine number and density for entire XII MN, and for soma, proximal dendrites and distal dendrites of mouse XII MNs

Age group (days)	Estimated total number of spines per XII MN	Soma counted spines (spine density as spines/100 μm^2^)	Proximal dendrites estimated spines (spine density as spines/100 μm)	Distal dendrites estimated spines (spine density as spines/100 μm)
E17–P0	412 ± 217	204 ± 212 (17.4 ± 25.3)	101 ± 53 (15.3 ± 12.4)	108 ± 98 (10.7 ± 7.8)
P1–P4	534 ± 271	260 ± 265 (22.1 ± 31.6)	140 ± 96 (15.1 ± 12)	135 ± 95 (10.4 ± 5.3)
P5–P8	1190 ± 407 ****E17–P0 ****P1–P4	464 ± 376 (29.5 ± 19.8)	368 ± 139 (33.8 ± 16.2) ****E17–P0, P1–P4	359 ± 300 (26 ± 13.7) **E17–P0, P1–P4, P9–P13
P9–P13	1017 ± 365 ***E17–P0 **P1–P4	230 ± 272 (17.4 ± 24.3)	158 ± 177 (8.7 ± 10.3) ***P5–P8	629 ± 214 (28.2 ± 9.2) ****E17–P0, P1–P4
P14–P28	1027 ± 609 ****E17–P0 ***P1–P4	345 ± 454 (15.4 ± 13.6)	232 ± 178 (14.5 ± 12) *E17–P0, P5–P8	450 ± 314 (22 ± 11.7) ****E17–P0, P1–P4

Mean ± SD. * *P* < 0.05, ** P < 0.01, *** *P* < 0.001, **** P < 0.0001 for one-way ANOVA with variable of age group, with Tukey’s multiple comparison post-test

A similar developmental pattern was seen for the number and density of spines located on proximal dendrites (Fig. 10i; Table 6). The average number of spines on proximal dendrites at E17–P0 increased slightly at P1–P4 and then more sharply at P5–P8, before decreasing at P9–P13 and then increasing again at P14–P28. As total spine counts will be influenced by changes in dendrite number and length, spine density per 100 μm of proximal dendrite length was determined. Proximal dendrite spine density was similar at E17–P0 and P1–P4, increasing markedly at P5–P8, and then decreasing at P9–P13 and P14–P28 (Table 6). However, in contrast to somatic spines, proximal dendrite spine numbers and spine density altered significantly with age group (one-way ANOVA, variable age group, *P* ≤ 0.0001, *F* > 10, *df* = 102; Fig. 10i; Table 6). Spine number on the proximal dendrites at P5–P8 was significantly elevated, compared to the E17–P0, P1–P4 and P9–P13 age groups (Fig. 10i; Table 6; Tukey’s multiple comparison test, *P* < 0.001, *q* > 6.5, *df* = 98 for all age group comparisons), and proximal spine number at P14–P28 was greater than at E17–P0 and P5–P8 (Tukey’s multiple comparison test, *P* < 0.05, *q* > 4.3, *df* = 98 for all age group comparisons).

The number of spines and spine density on distal dendrites also showed an increase with age (Fig. 10j; Table 6) up to P9–P13 and declined somewhat at P14–P28. The total number of spines on distal dendrites was highly influenced by age group (one-way ANOVA, variable age group, *P* < 0.0001, *F* > 15.2, *df* = 102 for both; Fig. 10j). The number of spines at both P9–P13 and P14–P28 were significantly increased compared to E17–P0, and P1–P4 age groups (Tukey’s multiple comparison, *P* < 0.0001, *q* > 6.8, *df* = 98 for all comparisons), and the P5–P8 age group was increased compared to E17–P0, P1–P4 and P9–P13 age groups (Tukey’s multiple comparison, *P* < 0.01, *q* > 4.8, *df* = 98 for all comparisons). In distal dendrites, the mean spine density was similar for E17–P0 and P1–P4 age groups, then increased markedly at P5–P8 and was maintained at similar levels at P9–P13 and P14–P28 (Table 6). The effect of age on distal dendrite spine density was also highly significant (one-way ANOVA, variable age group, *P* < 0.0001, *F* = 18.5, *df* = 102); distal spine density of P9–P13 and P14–P28 XII MNs was significantly greater than in both the E17–P0 and P1–P4 age groups (Tukey’s multiple comparison, *P* < 0.0001, *q* > 6.8, *df* = 98 for all comparisons) and spine density in the P5–P8 age group was significantly greater than in the E17–P0, P1–P4 and P9–P13 (Tukey’s multiple comparison, *P* < 0.01, *q* > 4.8, *df* = 98 for all comparisons).

#### Axons and axon collaterals

**Fig. 11:**
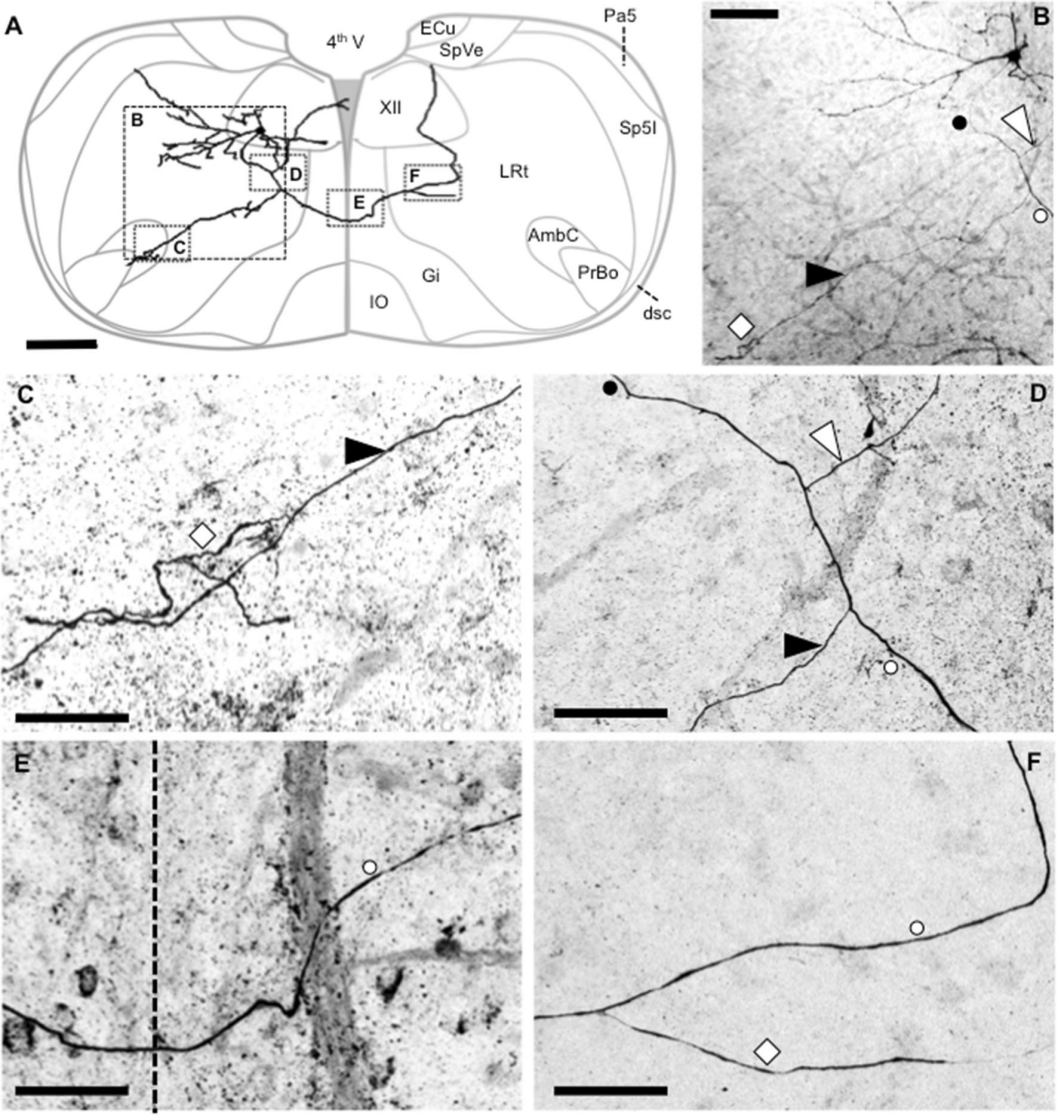
Axon and axon collaterals of a XII MN from a P10 mouse. **a** The flattened 2D computer reconstruction of the soma, dendrites, axon and axon collaterals of a single XII MN, projected onto a transverse brainstem section labeled with landmarks (*4th V* 4th ventricle, *AmbC* ambiguus compactus, *dsc* dorsal spinocerebellar tract, *ECu* external cuneate nucleus, *Gi* gigantocellular reticular nucleus, *IO* inferior olive, *Lrt* lateral reticular nucleus, *Pa5* paratrigeminal nucleus, *Sp5l* spinal trigeminal nucleus, interpolar part, *SpVe* spinal vestibular nucleus, *XII* XII motor nucleus); the areas outlined by the *dashed boxes* labeled **b**–**f** are shown as photomicrographs in panels **b**–**f**. **b** A low power confocal image of the XII MN depicted in **a**, showing the soma with an axon (*dark circle top right*) arising directly from it, which later gives rise to axon collaterals projecting ipsilaterally (*dark arrowhead*) and contralaterally (*white arrowhead* and *white circle*) within the area outlined in the *box* labeled **d**; the ipsilateral collateral branch has a terminal field (*white diamond*) within the area outlined in the *box* labeled **c**. **c** A high power confocal image of a portion of the ipsilateral axon collateral (labeled with *dark arrowhead*) within *box* **c**, which shows terminal branches (labeled with *white diamond*) in the region of AmbC**. d** A high power confocal image of the area within *box* **d**, showing ipsilateral (*dark arrowhead*) and contralateral (*white arrowhead* and *white circle*) axon collaterals arising from the main axon (*dark dot*). **e** A high power confocal image of the collateral marked with a *white circle* in **b** and **d** as the collateral crosses the midline (indicated by a *vertical black dashed line*). **f** A high power confocal image of the collateral marked with a *white circle* in **b** and **d**, and a further branch (marked with *white diamond*) in the reticular formation of the contralateral brainstem. *Scale bars* = 100 μm in **a**, **b**; and 25 μm in **c**–**f**

In 27 % (28 of 103) of filled XII MNs, an axon could be traced for at least 500 μm from its origin at either the soma (~80 % of MNs with an axon, 23/28 axons) or proximal dendrites (~20 % of MNs with an axon, 5/28 axons). By contrast, dendrites tapered, branched and displayed spines as they travelled away from the soma, whereas axons lacked branching (except as described below) and spines and showed a relatively constant diameter (mean of 1.1 μm, 95 % CI 0.99–1.12) as they projected away from the soma or proximal dendrite, usually in the ventral, ventrolateral or ventromedial directions (Fig. 11a, b). Occasionally axons initially projected in the dorsal direction and then turned around and travelled towards the ventrolateral medulla. Axons projected ventrolaterally on the ipsilateral side for variable distances before disappearing, presumably due to sectioning during brainstem slice preparation.

One of the most interesting findings of the present study was identification of axon collaterals in 21 % (6/28) of mouse XII MNs with filled axons (Fig. 11b, black and white arrowheads). This contrasts with several previous anatomical and functional studies, which have noted a morphological or functional absence of axon collaterals in XII MNs of several other species (Mosfeldt Laursen and Rekling 1989; Viana et al. 1990; Withington-Wray et al. 1988; Porter 1965). Cell bodies of XII MNs with axon collaterals were found throughout the hypoglossal motor nucleus. The origin of these collaterals in two XII MNs was less than 100 μm from the point of origin of the axon, occurring within the hypoglossal motor nucleus, while the other 4 XII MNs had collaterals arising 350–450 μm from the cell body, where the axon was outside the motor nucleus boundary. An example is shown in Fig. 11a, which is the flattened 3D reconstruction of a single XII MN, and Fig. 11b, which is a confocal image of the soma, proximal dendrites and axon of the same XII MN, with the main axon marked by a filled circle, and several collaterals by black and white arrowheads and a white circle. The main collateral axon trunk typically ran parallel to the axon (example not shown), although one cell had multiple collaterals that ran in a number of directions, including contralaterally (marked by white circle in Fig. 11c–f). Axon collateral projections to the contralateral brainstem were present in 2 XII MNs (see example from one XII MN shown in Fig. 11e, f). In five cells, the terminal field of axon collaterals was almost entirely outside the boundary of the hypoglossal motor nucleus, in the ipsilateral ventrolateral medulla (see example marked with white diamond in Fig. 11c) in the reticular formation, nucleus ambiguus compacts and pre-Bötzinger complex. A single XII MN had an axon collateral terminal field within the XII motor nucleus (not shown).

## Discussion

This study compares the morphology of single XII MNs from mice at ages from E17 to P28. The large number of XII MNs sampled, and their distribution throughout the entire somatotopically organized motor nucleus also allows a comparison of XII MNs that innervate different tongue muscles and shows that morphological properties of mouse XII MNs are relatively uniform for a given age, regardless of their position within the motor nucleus. However, many ventromedially located XII MNs have dendrites crossing the midline to arborize contralaterally at all ages. Developmental changes in morphology with increasing age are: (1) increased soma volume and surface, due to increased major axis length; (2) increased length, diameter and branch number for several dendrite branch orders; (3) decreased dye-coupling between XII MNs, and; (4) a distal shift in the dendritic location of spines. We also report for the first time axon collaterals in XII MNs, as ~20 % of XII MNs with filled axons had axon collaterals which arborized extensively in the ventrolateral medulla oblongata, and in one instance, within the XII motor nucleus. Due to the high proportion of XII MNs to interneurons in the XII nucleus (Viana et al. 1990, 1994), the large multipolar somata of XII MNs can be visually distinguished from smaller bipolar interneurons (Odutola 1976; Takasu and Hashimoto 1988). Critically, XII MN dendrites mainly extend in the transverse plane, allowing retention of complete individual MNs in transverse brain slices (Kanjhan and Bellingham 2013).

### Hypoglossal motor neurons in different parts of the motor nucleus show few morphological differences

To see whether any morphological differences correlated with the somatotopic organization of XII MNs innervating different tongue muscles in the mouse (Stuurman 1916), we divided the filled XII MNs into 3 groups, based on the location of their somata within the XII motor nucleus: dorsal, ventromedial and ventrolateral (Fig. 2a, b). This division was based on studies in the adult mouse and rat, showing somatotopic organization of XII MNs (Krammer et al. 1979; Altschuler et al. 1994; Aldes 1995; Stuurman 1916). The XII motor nucleus can be divided longitudinally into ventral and dorsal subnuclei (Krammer et al. 1979; Stuurman 1916). The styloglossus and the hyoglossus retractor muscles are innervated by MNs in the dorsal XII nucleus, while the geniohyoid and genioglossus protrude muscles are innervated by MNs in the ventrolateral and ventromedial portions of the XII nucleus, respectively (Stuurman 1916; Krammer et al. 1979; Aldes 1995; McClung and Goldberg 2002; Chamberlin et al. 2007). Our findings demonstrate that mouse XII MNs in these three divisions of the motor nucleus show few morphological differences, with one notable exception. XII MNs with dendrites that cross the midline are almost exclusively located in the ventromedial part of the motor nucleus, and thus are likely to be genioglossus MNs, based on their location and previous reports of retrogradely labeled genioglossus MNs having midline crossing dendrites (van Zundert et al. 2008; Núñez-Abades and Cameron 1995; Núñez-Abades et al. 1994; Altschuler et al. 1994).

### Morphological properties of XII MNs do not change between embryonic days 17–18 and postnatal day 0

The morphology of XII MNs before and after birth has not been compared in any species. Comparison of somatic volume, total and individual dendrite length and the density of spines on somatic, proximal dendrite and distal dendrites found no significant differences between XII MNs at E17–18 and P0. Our results show that mouse XII MNs have reached a morphological state prior to birth which enables the newborn to suckle, vocalize and maintain a patent upper airway at birth (Banks et al. 2005).

### Developmental changes in hypoglossal motor neuron somata

Mouse XII MN somata were ellipsoidal and relatively uniform in shape. Somata volume increased from approximately 3500 μm^3^ at birth to approximately 6000 μm^3^ at P9 and older, corresponding to a mean increase of 70 %. There was also a significant age-dependent increase in soma surface area and in the soma major axis, but not the minor axis, length. By contrast, rat genioglossus MNs showed no increase in soma surface area, or in major and minor ellipsoid axes from P1 to P30 (Núñez-Abades and Cameron 1995), while soma volume was not reported. Our values for soma surface area and the major/minor axes of mouse XII MNs are close to, but typically slightly lower than, those for rat genioglossus MNs of similar developmental stages (Núñez-Abades and Cameron 1995), implying that soma volume and surface area should also be similar and not changing with age. We used the formula for a prolate spheroid (Ulfhake and Cullheim 1988) to calculate soma volume from the mean major and minor axis diameter given in Núñez-Abades and Cameron (1995) and obtained soma volumes of 6100 (P1–2), 4600 (P5–6), 5800 (P13–15) and 7400 (P19–30) μm^3^ for rat genioglossus MNs. By contrast, cat spinal cord MNs show a soma surface area increase of approximately 100 % between P1 and P44 (Ulfhake and Cullheim 1988). However, the methodology of our study differs from others (Ulfhake and Cullheim 1988; Núñez-Abades and Cameron 1995); our soma surface area is directly calculated from the surface of a rendered 3D soma, while previous studies calculated surface area or volume for a prolate spheroid using major and minor axis length, which may potentially under- or over-estimate the actual cell volume of a complex 3D object (Bush and Sejnowski 1993). In particular, the presence of spines on the cell soma can significantly increase surface area and, to a lesser extent, volume (see below) (Bush and Sejnowski 1993).

### Ipsilateral dendritic projections and implications for synaptic inputs

XII MNs with dendrites extending beyond the XII motor nucleus border were mostly located at the caudal end of the motor nucleus, which contains XII MNs innervating genioglossus (ventromedial) or geniohyoid (ventrolateral) muscles (Aldes 1995; McClung and Goldberg 2002; Chamberlin et al. 2007). Dendrites extending beyond the XII nucleus border into the lateral reticular formation have been described in rat (Altschuler et al. 1994; Núñez-Abades et al. 1994; Koizumi et al. 2008) and in neonatal mice (Tarras-Wahlberg and Rekling 2009; van Zundert et al. 2008), consistent with our results. However, only 25 % of filled XII MNs had dendrites extending beyond the ipsilateral borders of the XII nucleus. Even within this sub-set of cells, only a small fraction (mean of 12 %) of the total dendritic length extended beyond the hypoglossal motor nucleus. Most extranuclear dendrites extended laterally, with only 5 % of filled cells having ventrolateral extranuclear dendritic extensions. Furthermore, we noted that spine density was less in distal dendrites outside the XII nucleus, when compared to spine density in distal dendrites that were within the nucleus.

Our findings suggest that only a small proportion of mouse XII MNs receive synaptic inputs outside the motor nucleus, and that XII MNs with dendrites extending beyond the border of the nucleus may have limited synaptic contacts on these extranuclear dendrites. It is likely that premotor excitatory and inhibitory synaptic inputs to XII MNs mainly terminate on XII MN dendrites within the hypoglossal motor nucleus. Inhibitory inputs onto distal dendrites extending ventrolaterally in the region of the Nucleus of Roller, are unlikely to participate in “shunting inhibition”, as this occurs on somata and primary dendrites (Marchetti et al. 2002; Berger 2011), consistent with glycine receptor and GABA_A_ receptor α1 subunit localization on the cell body and primary dendrites of XII MNs (O’Brien and Berger 2001; Muller et al. 2004; Fogarty et al. 2013; Liu and Wong-Riley 2006, 2013).

If most of the XII MN dendritic tree is within the XII nucleus, any interneurons within the XII nucleus, although not numerous, is likely to provide significant inputs to XII MNs. During these experiments, we have also filled several small interneurons within the XII nucleus of mice; these bipolar cells have small somata with a simple dendritic tree, and have axons with extensive arborization within the XII nucleus, forming close contacts with the dendrites of adjacent XII MNs also filled with Neurobiotin (R. Kanjhan, unpublished observations). These neurons may be glycinergic interneurons, as interneurons expressing glycine transporters are scattered within the XII nucleus or laterally in the border area between the XII nucleus and the surrounding lateral medullary reticular formation in the rat (Tanaka et al. 2003), while GABAergic interneurons are relatively uncommon within the XII nucleus, occurring mainly in the ventrolateral Nucleus of Roller (van Brederode et al. 2011).

Synaptic inputs undergo significant modification due to passive cable properties of dendrites (Gustafsson and Pinter 1984; Spruston et al. 1994; Rall 1959; Jack and Redman 1971), before integration at the axon initial segment and action potential generation (Kole et al. 2008; Kole and Stuart 2012). Passive cable properties also markedly attenuate voltage control imposed at the soma (Spruston et al. 1993, 1994; Williams and Mitchell 2008). Our morphological measurements will allow the generation of equivalent cylinder models (Bush and Sejnowski 1993) for XII MNs during dendritic development (Fig. 8e). As Fig. 8e shows, the development of the equivalent cylinder structure of mouse XII MNs occurs in two distinct phases, using the XII MN model at E17–P0 as a baseline. Firstly, from P1 to P4, there is an increase in length of the 1st, 2nd, 3rd and higher branch order cylinders, which are then relatively stable during P5–P8. Secondly, from P9 to P13, a marked increase in length of the higher branch order equivalent cylinders occurs, accompanied by smaller increases in length for 1st and 2nd order equivalent cylinders; during P14–P28, 1st, 2nd and higher order cylinder length is relatively stable, while 3rd order cylinder length and higher order cylinder diameter increase. In the functionally and morphologically mature XII MN (P14–P28), higher order dendrites comprise approximately 50 % of the total dendritic cylinder length. As the marked increase in spine density in distal dendrites later in development implies a distal shift in the site of synaptic inputs, this change in the dendritic equivalent cylinder means that these inputs become more electrotonically remote, and will be heavily filtered as they propagate along the dendrites to the soma and axon initial segment.

A second consequence is that effective voltage control of the majority of synapses onto mature XII MNs is not possible from a somatic site. Direct measurements of somatic voltage control in apical dendrites of cortical pyramidal neurons has shown that voltage control of fast synaptic events rapidly attenuates as the synaptic input moves distally; at 50–100 μm from the soma, only 50–70 % of the synaptic current is recorded by a somatic voltage clamp, while at more than 180 μm from the soma, there is effectively no voltage control (Williams and Mitchell 2008; Spruston et al. 1993). In rhythmically active rodent XII MNs from P0 to P21, glutamatergic inspiratory synaptic drive produces relatively small changes in membrane conductance at the soma (Funk et al. 1997b; Ramirez et al. 1996). It is therefore likely that glutamatergic inputs producing the inspiratory drive to XII MNs are predominantly distal, as the length of the 1st order dendritic cylinder length never exceeds 100 μm in XII MNs, and a somatic electrode should be able to record and partially voltage-clamp increases in membrane conductance due to synaptic inputs on soma and primary dendrites. We also note that voltage control of slowly changing ionic currents such as I_H_ or delayed rectifier potassium currents extends much further into the dendritic cylinder (Spruston et al. 1993).

### Contralateral dendritic projections

Overall, 26 % of all Neurobiotin-filled XII MNs had dendrites crossing the midline (see example of crossings in Fig. 9b). All but 3 of such cells were located at the ventromedial subnucleus likely to contain genioglossus MNs. Of the cells located in the ventromedial XII nucleus, the proportion with contralateral dendrite projections was 42 % (*n* = 45). Contralateral dendrite projections declined with age, but were found in all age groups. The persistence of contralateral dendrites of mouse XII MNs between E17 and P28 differs from previous findings in the rat (Altschuler et al. 1994; Núñez-Abades et al. 1994; Wan et al. 1982; Koizumi et al. 2008), where contralateral dendrites were not seen between P8 and P18 (Núñez-Abades et al. 1994). Retrograde labeling of multiple XII MNs have also revealed contralateral dendrites in mice at P0–P5 (Tarras-Wahlberg and Rekling 2009), and P6 but not at P9 (van Zundert et al. 2008). Thus, in the mouse, unlike in the rat, contralateral dendrites are maintained through development to adulthood, although the % of XII MNs with contralateral dendrites declines progressively during development.

XII MN dendrites are not unique in crossing the midline to arborize in the contralateral CNS, as this is seen in other MN pools innervating axial or respiratory muscles, including neonatal rat phrenic (Lindsay et al. 1991), cervical (Rose 1981; Rose and Richmond 1981), intercostal (Lipski and Martin-Body 1987) and sacral/coccygeal MNs (Jankowska et al. 1978; Light and Metz 1978; Ritz et al. 1992; Rand and Breedlove 1995). However, a small sample of cat XII MNs did not show contralateral dendrites (Fukunishi et al. 1999), suggesting that there may be species differences in XII dendrite projection patterns. The functional role of XII MN contralateral dendrites may be to facilitate synchronized bilateral muscle activity. XII MNs with contralateral dendrites may be a sub-population of MNs receiving input from rhythmically active contralateral pre-Bötzinger Complex neurons (Koizumi et al. 2008; Thoby-Brisson et al. 2009; Morgado-Valle et al. 2010; Tarras-Wahlberg and Rekling 2009), as axons of last order premotor interneurons do not cross the midline.

### Dye-coupling between XII MNs

Dye-coupling between mouse XII MNs was common (38 % of Neurobiotin-filled XII MNs) at or shortly after birth. However, the presence of dye-coupling in XII MNs declined over the first postnatal week and was never seen in P14–P28 XII MNs. It has been reported that 15–42 % of rat XII (Mazza et al. 1992), phrenic (Martin-Caraballo and Greer 1999) and lumbar (Walton and Navarrete 1991; d’Incamps et al. 2012) MNs are electrotonically coupled during embryonic and neonatal periods. Our study suggests that the rate of coupling is higher in XII MNs (up to 42 %) compared to that seen in lumbar and phrenic MNs (15–25 %). Dye-coupled XII MNs were always ipsilateral to each other, and showed close anatomical proximity. This suggests that dye-coupled XII MNs are likely to innervate the same muscle, consistent with electrotonic coupling between adjacent developing rat lumbar MNs being restricted to those MNs innervating the same muscle (Walton and Navarrete 1991). In several instances, the only close contacts between dye-coupled XII MNs were between dendrites (e.g., Fig. 9c, d), suggesting that direct dendro-dendritic interactions between XII MN processes may occur, as seen for other brainstem MNs with dendro-dendritic contacts (Bras et al. 1987).

Electrotonic coupling is thought to facilitate sharing of currents and/or intracellular messengers that may be involved in controlling intercellular communication, neurogenesis, differentiation, and/or circuit formation (Montoro and Yuste 2004; Sutor and Hagerty 2005; Elias and Kriegstein 2008; Connors and Long 2004). Previous studies in neonatal preparations have demonstrated that synchronized rhythmic motor activity may be partly due to gap junctions (Tresch and Kiehn 2000, 2002; Kiehn and Tresch 2002). Although gap junction proteins (pannexins and connexins) are found in adult spinal interneurons and MNs (Rash et al. 2000; van der Want et al. 1998), their functional role in neuronal communication in adult animals is not well established (Chang et al. 2000). Neighboring adult phrenic motor neurons and their primary dendrites are progressively separated by astrocytic processes during development (Goshgarian and Rafols 1984). Altering local astrocytic processes (e.g., spinal cord injury) can result in a significant increase in the number of dendro-dendritic appositions between adult spinal MNs (Goshgarian et al. 1989; Sperry and Goshgarian 1993). Hemi-channels in adult MNs may also function to release signaling molecules, such as ATP (Hansen et al. 2014; Connors and Long 2004). We suggest that, although dye-coupling declines with maturation, dye-permeable gap junctions persist as latent hemi-channels and might assume an important function following stimuli which induce long term facilitation of XII MN discharge, such as repetitive/hypoxic stimulation of MNs (Bach and Mitchell 1996; Fuller et al. 2001).

### The functional role of contralateral dendrites and MN dye-coupling

Breathing is a bilaterally synchronous motor behavior that relies on the respiratory rhythm generator formed by the pre-Bötzinger Complex in the ventrolateral medulla oblongata (Smith et al. 1991). Synchronization of MN firing during prenatal and neonatal development is thought to involve midline crossing of dendrites and electrotonic coupling (Tresch and Kiehn 2000; Chang and Balice-Gordon 2000; Tarras-Wahlberg and Rekling 2009). Bilateral coordination of breathing after birth is vital, as shown by Robo3 null mutant mice, which lack midline crossing of Pre-Bötzinger Complex neuron and interneuron axons in the hindbrain and spinal cord, and show desynchronized breathing and death shortly after birth (Bouvier et al. 2010). The midline dendrite crossings in mouse are therefore likely to be involved in bilateral coordination of genioglossus MNs and inspiratory muscle activity, in addition to direct midline coupling of pre-Bötzinger Complex neurons (Tarras-Wahlberg and Rekling 2009).

### Spine distribution and density

Santiago Ramón y Cajal first described dendritic spines in Golgi studies of Purkinje neurons (Ramón y Cajal 1909). Since then, many studies have characterized the morphology and functions of dendritic spines, primarily for hippocampal, cortical and cerebellar neurons (Harris and Weinberg 2012; Kanjhan et al. 2015). These studies show that dendritic spines are dynamic sites where excitatory synapses are formed (Harris et al. 1992; Engert and Bonhoeffer 1999). A long-held assumption is that all spines receive excitatory synaptic inputs, and a recent serial reconstruction of spines in adult mouse cortical neurons found that <5 % of spines were non-synaptic, with these being filopodia-like in structure (Arellano et al. 2007).

Although some studies reported spines or micro-dendrites on MN dendrites (Rose and Richmond 1981; Yoshida et al. 1987; Destombes et al. 1983; Bras et al. 1987; Cameron et al. 1985; Goshgarian and Rafols 1981; Odutola 1976), only a few quantitative descriptions of spines or their potential roles in MNs exist (Ma and Vacca-Galloway 1991; Bou-Flores et al. 2000; Takashima et al. 1990; Bras et al. 1987; O’Kusky 1998; Kanjhan et al. 2015). Ultrastructural studies of hypoglossal and other brainstem MNs show fine (<1 μm diameter) micro-dendrites emerging from somatic and dendritic membranes and receiving synaptic inputs (Bras et al. 1987; Destombes et al. 1983; O’Kusky 1998). An electron microscopy study of synaptic contacts in the developing rat hypoglossal motor nucleus (O’Kusky 1998) is of particular interest. This study found a ~2:1 ratio of asymmetric (excitatory) and symmetric (inhibitory) synapses (Gray 1959) at all developmental ages, with both types of synapse progressively shifting towards dendritic membrane during development to P30 (O’Kusky 1998). However, while both asymmetric and symmetric synapse were found on dendritic spines, peak levels (~7 %) of asymmetric synapses on spines occurred at P5, similar to the transient increase in somatic and proximal dendrite spine density seen at P5–8 in mouse XII MNs, while symmetric synapses on spines was maximal at birth (2 % of symmetric synapses) and declined progressively with age to >0.5 % of symmetric synapses (O’Kusky 1998). It thus seems likely that the spines we have observed on mouse XII MNs receive synaptic inputs, which are predominantly excitatory (Fogarty et al. 2013).

With our dye-filling method, we see developmental changes in the density and location of spines on the soma and dendrites of mouse XII MNs (Kanjhan et al. 2015). The spine density is significantly higher at the soma and primary dendrites in late embryonic and neonatal XII MNs, with spines starting to appear at distal dendrites at P5 and then gradually increasing in density in the distal dendrites over the next 3 weeks, consistent with electron microscopic studies of synapse distribution in rat hypoglossal motor nucleus (O’Kusky 1998). While the presence of a spine does not unequivocally correspond to the presence of a synapse, there is a similarity between the spine density (9–28 spines per 100 μm of dendrite) seen here, and the density of ultrastructurally characterized synapses (9–15 boutons per 100 μm) on the dendritic membrane of other MNs (Bras et al. 1987; O’Kusky 1998). This is likely to be of functional significance, as spines are thought to amplify synaptic currents generated by contacting synapses, due to the relatively greater input resistance of the spine structure compared to that of somatic or dendritic membrane (Bras et al. 1987; Gulledge et al. 2012; Harnett et al. 2012). A second consequence of synaptic input onto spines is that somatic measurement of synaptic conductance changes in spines will be attenuated, consistent with reports that endogenous activation of non-NMDA glutamate receptors during rhythmic depolarization produces relatively small changes in membrane conductance measured at the soma (Funk et al. 1997b; Ramirez et al. 1996). As two major developmental changes seen in mouse XII MNs were the elongation of distal dendrite compartments and a distal shift in spine location, there is likely to be an increasing mismatch between the inspiratory synaptic conductance at the soma and the actual conductance change due to distal synaptic inputs during development in mice. Finally, the presence of additional neuronal membrane with its passive leak conductance in spines results in increased total membrane capacitance and decreased neuronal input resistance, which need to taken into account for computational models of neurons (Bush and Sejnowski 1993; Stuart and Spruston 1998).

### Axon collateral projections

Previous studies have reported that XII MNs do not have axon collaterals (Koizumi et al. 2008; Núñez-Abades et al. 1994; Withington-Wray et al. 1988; Ramón y Cajal 1909; Porter 1965; Viana et al. 1990; Mosfeldt Laursen and Rekling 1989). It was therefore surprising to find that in 28 mouse XII MNs with axonal labeling (at least 500 μm of filled axon), approximately 20 % (6/28) also had axon collaterals. Two factors may account for this observation. Firstly, our study had sufficient sampling power to reveal this morphological feature (103 MNs in total, with 28 having filled axons filling, and 6 of these having axon collaterals), compared to previous studies which sampled 10, 40 and 7 MNs, respectively (Koizumi et al. 2008; Núñez-Abades et al. 1994; Withington-Wray et al. 1988). Secondly, we sampled XII MNs throughout the XII nucleus, while XII MNs in previous studies were confined to the ventromedial genioglossus area of the XII nucleus (Koizumi et al. 2008; Núñez-Abades et al. 1994; Withington-Wray et al. 1988). The target field of these XII MN axon collaterals was largely ipsilateral and outside the XII nucleus, extending laterally and ventrally (Fig. 11) to areas containing premotor neurons (Dobbins and Feldman 1995; Peever et al. 2002; Chamberlin et al. 2007; Koizumi et al. 2008). This projection pattern suggests that these axon collaterals could provide a source of feedback control for inspiratory synaptic drive. Other respiratory MN pools, such as laryngeal MNs and other MNs of the nucleus ambiguus compactus, are located in this region and receive cholinergic inputs (Bautista et al. 2010), suggesting that XII MN axon collaterals might act to coordinate motor activity in different MN pools. Indeed, XII MNs themselves receive cholinergic synapses (Connaughton et al. 1986; Bautista et al. 2010). Taken together with our observation that one axon collateral terminated extensively within the XII motor nucleus, it is possible that postsynaptic nicotinic (Chamberlin et al. 2002; Shao et al. 2008; Liu et al. 2005) and muscarinic (Ireland et al. 2012) responses of XII MNs might be activated by recurrent collateral inputs from other XII MNs.

XII MN development occurs during a period of significant functional changes in respiration, feeding and synaptic plasticity in rodents (Kubin and Volgin 2008). Our data show that dendrite length and spine density increases up to P8 and then largely stabilizes, but with spinogenesis shifting from proximal to distal dendritic compartments, consistent with postnatal changes of key excitatory and inhibitory neurotransmitter receptor expression (Gao et al. 2011; Liu and Wong-Riley 2005, 2006, 2010, 2013). As XII MNs strongly express TrkB (Thoby-Brisson et al. 2003), this developmental plasticity may be driven by brain derived neurotrophic factor (BDNF), which activates TrkB, enhancing dendrogenesis and synaptogenesis and which is critical for normal postnatal development of respiration (Erickson et al. 1996; Thoby-Brisson et al. 2003; Kron et al. 2007a, b, 2008).

## Conclusion

In conclusion, these data have shown that mouse XII MNs have a relatively similar morphological structure despite innervating functionally different tongue muscles. The presence of dendritic spines and axon collaterals expand the possible ways that XII MNs can receive and process synaptic inputs and participate in brainstem circuitry output. The changes in morphological structure of individual mouse XII MNs during development to maturity are complex and are likely to have a significant impact on the integration of synaptic inputs required for the regulation of XII MN firing needed to carry out several basic and vital motor activities. One way forward would be the use of these detailed morphological structures in biophysical computer models (Purvis and Butera 2005) to develop a better understanding of how interactions between synaptic information and intrinsic ion conductances are ultimately translated into XII MN firing patterns.
